# Pharmacological Insights into *Codonopsis lanceolata*: A Review of Its Potential in Disease Prevention and Therapy

**DOI:** 10.3390/molecules31132327

**Published:** 2026-07-02

**Authors:** Sanjay, Rachit Sood, Hae-Jeung Lee

**Affiliations:** 1 Department of Food and Nutrition, College of BioNano Technology, Gachon University, Seongnam 13120, Gyeonggi-do, Republic of Korea; 2 Institute for Aging and Clinical Nutrition Research, Gachon University, Seongnam 13120, Gyeonggi-do, Republic of Korea; 3 Department of Health Sciences and Technology, Gachon Advanced Institute for Health Science and Technology (GAIHST), Gachon University, Incheon 21999, Republic of Korea; 4 Gachon Biomedical Convergence Institute, Gachon University Gil Medical Center, Incheon 21565, Republic of Korea

**Keywords:** *C. lanceolata*, antioxidant, anti-inflammatory, diabetes, cancer, cardiovascular disease, Deodeok

## Abstract

*Codonopsis lanceolata* has long been used in traditional medicine across East Asia; however, recent scientific investigations have increasingly highlighted its broad therapeutic potential. This review provides an updated and comprehensive overview of the pharmacological properties of *C. lanceolata* and its bioactive constituents, focusing on their roles in disease prevention and therapy. Evidence from in vitro, in vivo, and limited clinical studies suggests that *C. lanceolata* possesses antidiabetic, anti-obesity, and anti-cancer properties while showing protective effects in experimental models of cardiovascular dysfunction, liver injury, neurodegenerative disorders, skeletal muscle atrophy, and pulmonary damage. Additionally, its immunomodulatory effects contribute to improved host defense and regulation of inflammatory responses. These diverse actions are mediated by mechanisms involving antioxidant activity, inhibition of inflammatory signaling, regulation of metabolic pathways, modulation of apoptosis, and maintenance of tissue integrity. Despite promising findings, challenges remain regarding extract standardization, identification of key active compounds, and the translation of preclinical results into clinical efficacy. Future research integrating molecular, pharmacokinetic, and clinical approaches is essential to clarify the therapeutic value and establish its potential as a nutraceutical or therapeutic agent. This review highlights the promising pharmacological potential and identifies key directions for future research to support its potential application in modern medicine.

## 1. Introduction

The genus *Codonopsis* (family Campanulaceae) comprises approximately 40 species, many of which are distributed across East and Southeast Asia, including China, Korea, and Japan. The roots of *C. pilosula* var. *modesta* and *C. tangshen* have been reported to possess immune system-enhancing properties, improve gastrointestinal function, attenuate gastric ulcers, stimulate appetite, and reduce blood pressure and are used as traditional medicines for the treatment of various diseases in Japan, China, and Vietnam [1]. Additionally, *Codonopsis* species are used as ingredients in tea, wine, and soups [2,3].

**Figure 1:**
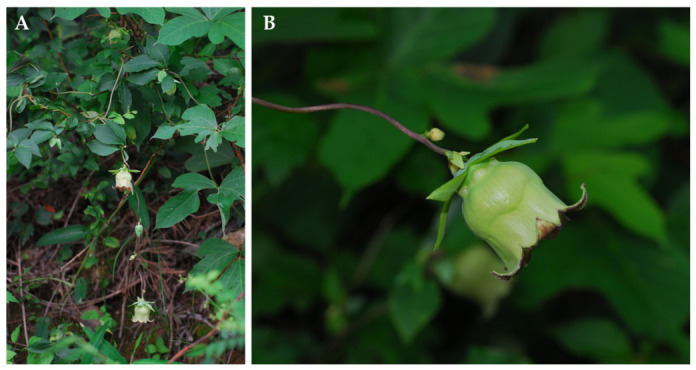
Morphological characteristics of *C. lanceolata*. (**A**) *C. lanceolata* in its natural environmental habitat, displaying its climbing stem, leaves, and pendulous bell-shaped flowers. (**B**) Detailed view of a typical bell-shaped flower of *C. lanceolata*. Photographs provided courtesy of the Korea National Arboretum (Field Expert Sa-Beom Jang).

Among various species of *Codonopsis*, *C. lanceolata*, commonly known as bonnet bell flower, “deodeok” in Korea, and “shashen” in China, has emerged as one of the most extensively studied species owing to its longstanding use in traditional medicine and its diverse pharmacological potential [4]. *C. lanceolata* is a perennial herb that grows naturally in moist areas, such as forests, low mountains, and hilly regions, reaching heights of up to 1.5 m. It is widely distributed across East Asian countries, including China, Korea, and Japan [5,6]. The flowering season extends from August to September, and the flowers are bell-shaped, purple, hermaphroditic, and pollinated by wasps and bees. Seeds ripen from September to October. The plant thrives in full sun to semi-shade, light, and medium- to well-drained soils with adequate moisture and neutral-to-acidic soil conditions. The roots of *C. lanceolata* have long been used in food and traditional medicine, and the plant is widely cultivated in home gardens in South Korea [4]. The morphological characteristics of *C. lanceolata* are shown in Figure 1. Ethnopharmacological records indicate that *C. lanceolata* has traditionally been used to treat respiratory ailments such as asthma and bronchitis, chronic fatigue, digestive disturbances, and inflammatory disorders [7]. These traditional applications have been attributed to the plant’s anti-inflammatory and antioxidant properties and their ability to mitigate oxidative stress (OS).

**Figure 2:**
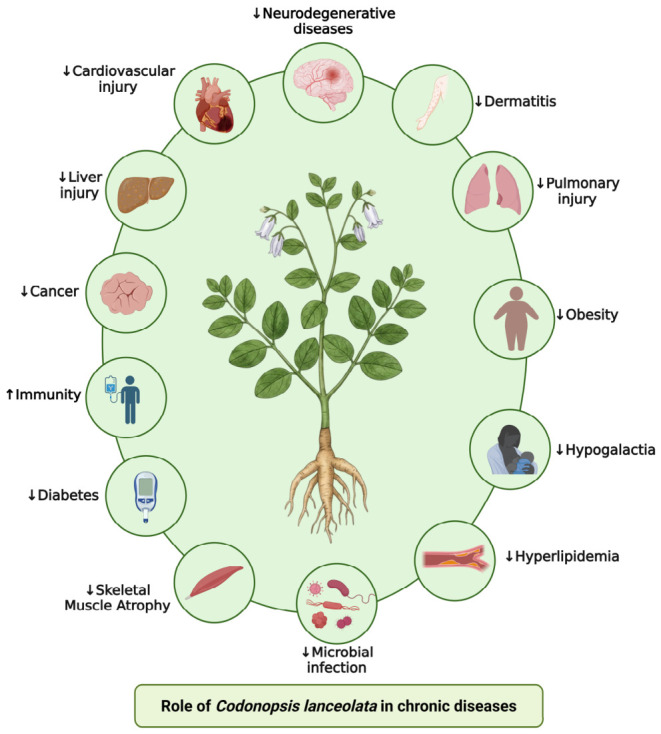
Therapeutic potential of *Codonopsis lanceolata* in the prevention and management of chronic diseases. The figure summarizes the pharmacological roles of *C. lanceolata*, showing its potential to mitigate various conditions, including cardiovascular injury, liver injury, pulmonary injury, neurodegenerative diseases, dermatitis, cancer, obesity, diabetes, hyperlipidemia, and skeletal muscle atrophy, while also acting to combat microbial infection and enhance immunity, with a specific mention of its traditional use in managing hypogalactia. ↑, increase; ↓, decrease. Created in BioRender. Lee, H. (2026) https://BioRender.com/ifjxatu (accessed on 5 June 2026).

Modern phytochemical investigations have revealed that *C. lanceolata* contains diverse secondary metabolites, including triterpenoid saponins, polysaccharides, phenolic compounds, flavonoids, alkaloids, and phenylpropanoid glycosides. Among these metabolites, lancemasides and related triterpenoid saponins are considered characteristic constituents of the plant and are believed to contribute substantially to its pharmacological activities [8,9,10,11,12,13,14,15]. The detailed phytochemical composition and biological relevance of these metabolites are discussed in Section 3. Accumulating evidence indicates that *C. lanceolata* and its secondary metabolites may contribute to the amelioration of a wide range of chronic diseases by modulating OS, inflammatory signaling, immune responses, and metabolic homeostasis, suggesting potential protective effects against cardiovascular, metabolic, neurodegenerative, and organ-specific disorders (Figure 2).

Besides its pharmacological properties, *C. lanceolata* is also valued for its nutritional composition; it contains essential amino acids, vitamins, and minerals that enhance its function as a functional food [16]. The plant is cultivated under specific agronomic conditions to preserve the concentration of its active constituents, and ongoing research continues to optimize cultivation techniques to improve yield and bioactive compound content [17]. With growing interest in natural products and plant-derived therapeutics, *C. lanceolata* has become a focal point for studies aimed at integrating traditional ethnomedicinal knowledge with modern biomedical research. Such integrative approaches not only deepen our understanding of the therapeutic potential of *C. lanceolata* but also pave the way for the development of novel drugs, dietary supplements, and nutraceuticals. This review provides an updated overview of the phytochemistry, pharmacological activities, molecular mechanisms, and therapeutic potential of *C. lanceolata*, highlighting the current research gaps and future directions for its development as a functional food, nutraceutical, and therapeutic agent.

## 2. Literature Search Strategy

This review aimed to explore the current scientific literature on the pharmacological potential of *Codonopsis lanceolata* in the prevention and treatment of various chronic diseases. A literature search was carried out using Google Scholar, PubMed, and Science Direct repositories for related findings between January 2001 and February 2025. The following keywords were used to find the relevant information regarding the anti-inflammatory and antioxidant role of *C. lanceolata*: “*Codonopsis lanceolata* and its anti-inflammatory properties” or “*Codonopsis lanceolata* in oxidative injuries” or “*Codonopsis lanceolata* in neurodegenerative disorders” or “*Codonopsis lanceolata* in diabetes” or “*Codonopsis lanceolata* in metabolic disorders” or “*Codonopsis lanceolata* in cardiovascular disorders” or “*Codonopsis lanceolata* in pulmonary dysfunction” or “*Codonopsis lanceolata* in obesity”. The search was limited to peer-reviewed articles published in English between 2001 and 2025. In total, 101 publications were included in this review, comprising 72 research articles and 29 review papers. Studies were selected based on relevance, with the inclusion criteria focusing on experimental, clinical, and mechanistic studies examining *Codonopsis lanceolata* and its bioactive compounds in the context of OS and inflammation-associated chronic diseases. Duplicate, non-English, and unrelated studies were excluded.

## 3. Phytochemical Profile and Bioactive Constituents of *Codonopsis lanceolata*

*Codonopsis lanceolata* contains a diverse range of phytochemicals that are believed to underlie its pharmacological activity. Various plant parts, particularly the roots, have been extensively investigated and shown to contain bioactive compounds, including triterpenoid saponins, polysaccharides, phenolic compounds, flavonoids, and other secondary metabolites [1,13,18,19]. Among these constituents, triterpenoid saponins are the most characteristic compounds reported in *C. lanceolata* [1,13]. Several saponins, including lancemaside A, lancemaside B, lancemaside C, and lancemaside D, have been isolated and characterized from plants, with lancemaside A being one of the most extensively studied [1,11]. In addition to lancemasides, other triterpenoid saponins, including codonolaside and oleanane-type saponins, have been identified in *C. lanceolata* roots [13,14]. These compounds have been reported to exhibit various biological activities, including anti-inflammatory, antioxidant, immunomodulatory, and anti-cancer effects [10,20]. The traditional medicinal uses of *C. lanceolata*, including the management of respiratory and gastrointestinal disorders such as cough, bronchitis, and colitis, have been suggested to be partly associated with lancemaside A and its metabolites, particularly echinocystic acid [4,12].

In addition to saponins, *C. lanceolata* contains various other polysaccharides that contribute to its pharmacological properties. Plant-derived polysaccharides have immunomodulatory, antioxidant, and metabolic effects. Studies investigating *C. lanceolata* polysaccharides have demonstrated their ability to improve glucose metabolism, enhance antioxidant defense systems, and regulate inflammatory responses [21,22]. These polysaccharides are important contributors to the pharmacological activities of plants [22]. Recent studies have suggested that *C. lanceolata* polysaccharides exert their biological activities by modulating OS-related pathways and cytokine-mediated signaling networks, thereby contributing to systemic metabolic homeostasis [21,22].

Phenolic compounds and flavonoids constitute an important group of phytochemicals found in *C. lanceolata* [15,16]. These compounds are widely recognized for their antioxidant capacity and ability to neutralize reactive oxygen species (ROS) [16]. Phenolic constituents, such as phenylpropanoid glycosides and other related metabolites, have been identified in *C. lanceolata* extracts and are associated with neuroprotective, hepatoprotective, and cardioprotective effects [23,24,25]. Recent phytochemical investigations have identified chlorogenic acid, tangshenoside derivatives, and other phenolic metabolites as major contributors to the antioxidant capacity of *C. lanceolata* extracts [15,16]. The presence of these compounds significantly contributes to the plant’s ability to mitigate OS-related cellular damage.

Furthermore, additional bioactive molecules such as tangshenosides, echinocystic acid, and other triterpenoid derivatives have been reported in *C. lanceolata* [5,12,15,18,26]. These compounds exhibit multiple biological activities, including modulation of metabolic pathways, regulation of apoptosis, and inhibition of inflammatory signaling [26,27,28,29,30]. The complex phytochemical composition of *C. lanceolata* suggests that its reported pharmacological activities may arise, at least in part, from interactions among multiple bioactive constituents, rather than from a single active molecule [1,3,4].

The phytochemical composition of *C. lanceolata* varies considerably depending on the plant part, geographical origin, cultivation conditions, processing methods, and extraction procedures. Although the roots remain the most extensively studied medicinal part and contain abundant triterpenoid saponins, phenylpropanoid glycosides, and polysaccharides, recent metabolomic analyses have identified distinct phenolic and flavonoid profiles in the aerial tissues and sprouts, suggesting that different plant parts may contribute to unique biological activities [1,16,17,18,29]. In addition, extraction methods, including water extraction, ethanol extraction, fermentation, and high-pressure processing, have been shown to influence both the qualitative and quantitative compositions of phytochemicals, thereby affecting their biological activities [19,29,30,31,32,33]. These findings highlight the importance of phytochemical standardization and quality control to ensure reproducible pharmacological efficacy and facilitate the future clinical translation of *C. lanceolata*-based products.

Collectively, the rich phytochemical profile of *C. lanceolata* provides the biochemical basis for its wide range of pharmacological activities. Understanding the chemical constituents of this plant is essential for elucidating the molecular mechanisms underlying its therapeutic potential and facilitating the development of standardized extracts and nutraceutical formulations.

## 4. Pharmacological Potential of *Codonopsis lanceolata* Across Disease Models

The traditional medicinal plant *Codonopsis lanceolata* (Deodeok) has garnered significant interest for its potential to manage chronic diseases, which is largely attributable to its rich composition of bioactive compounds. Various parts of the plant, including the roots, stems, leaves, and flowers, contain secondary metabolites and other phytochemical constituents, such as saponins, polysaccharides, and phenolic compounds. Following extraction and metabolism, these compounds have been reported to modulate several biological processes that may contribute to the pharmacological activities of the plant. Specifically, *C. lanceolata*-derived compounds have been reported to modulate cellular processes by enhancing phagocytosis, apoptosis, and glucose uptake while reducing OS, inflammation, and adipogenesis in experimental models.

**Figure 3:**
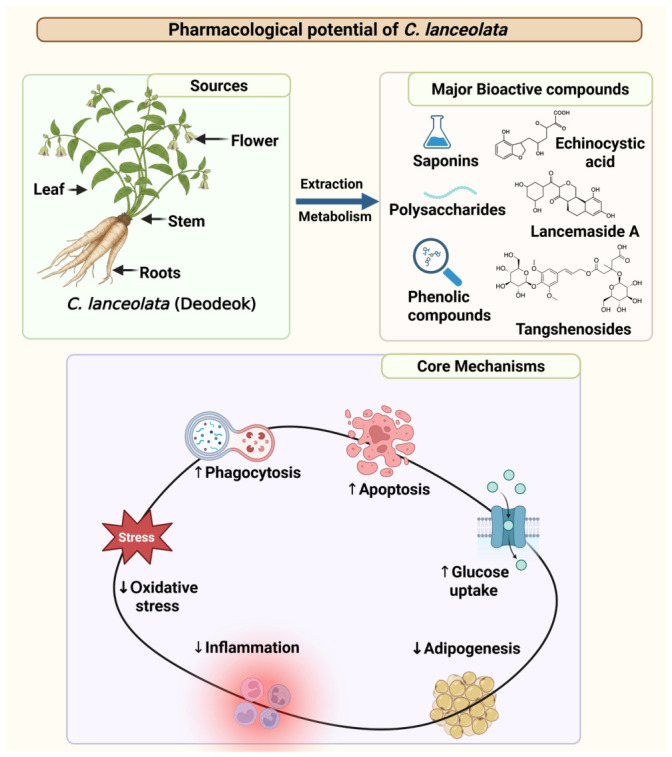
Pharmacological potential of *Codonopsis lanceolata* (Deodeok). The plant’s roots, stems, leaves, and flowers serve as sources for bioactive compounds, including saponins, polysaccharides, and phenolic compounds, which can be extracted and metabolized. These compounds may exert core mechanisms of action by enhancing phagocytosis, apoptosis, and glucose uptake while reducing oxidative stress, inflammation, and adipogenesis, collectively contributing to stress mitigation and overall pharmacological effects.↑, increase; ↓, decrease. Created in BioRender. Lee, H. (2026) https://BioRender.com/ifjxatu (accessed on 5 June 2026).

However, the pharmacological effects of *C. lanceolata* are influenced by the extraction and purification methods used. Previous studies have employed a wide range of preparations, including aqueous, methanolic, and ethanolic extracts of varying concentrations; solvent fractions, such as ethyl acetate and n-butanol; isolated polysaccharides; and purified compounds, including lancemaside A, echinocystic acid, and tangshenoside I. These methods enrich different groups of bioactive constituents selectively, resulting in distinct phytochemical compositions and diverse biological activities. As a result, differences in the therapeutic outcomes reported among studies may, at least in part, be attributed to variations in extract composition. Therefore, direct comparisons among studies should be performed with caution. Nonetheless, the overall body of evidence indicates that *C. lanceolata* possesses diverse pharmacological properties that may alleviate cellular stress and modulate multiple pathological processes involved in chronic diseases (Figure 3).

### 4.1. Diabetes and Obesity

OS plays a pivotal role in the development and progression of diabetes and obesity [34]. An imbalance between ROS production and antioxidant defense leads to cellular damage, inflammation, and metabolic dysfunction [35]. In obesity, excessive fat accumulation increases ROS generation from the adipose tissue and mitochondria, which in turn promotes insulin resistance [36]. Similarly, in diabetes, chronic hyperglycemia enhances OS through glucose auto-oxidation and activation of oxidative pathways, further impairing insulin signaling and pancreatic β-cell function [37]. Together, these processes create a vicious cycle that links OS to the onset and complications of metabolic diseases, such as obesity and diabetes.

Lee et al. investigated and compared the antioxidant and antiadipogenic activities of five wild herbs: *Ligularia fischeri*, *Aster scaber*, *Kalopanax pictus*, *C. lanceolata*, and *Cirsium setidens*. The *Ligularia fischeri* showed the highest total phenolic content (TPC, 215.8 ± 14.2 mg GAE/g), followed by *Aster scaber*, *Kalopanax pictus*, *Cirsium setidens*, and *C. lanceolata*, while *Aster scaber* showed the highest total flavonoid content (TFC, 103.9 ± 3.4 mg RE/g). All extracts exhibited dose-dependent antioxidant activity, as evidenced by their 2,2-diphenyl-1-picrylhydrazyl (DPPH) and 2,2′-azino-bis-(3-ethylbenzothiazoline-6-sulfonic acid) (ABTS) radical scavenging capacities, indicating effective free radical neutralization. Ferric reducing antioxidant power (FRAP) and reducing power assays further demonstrated their potent electron-donating capacity, reflecting enhanced redox potential. Additionally, the high oxygen radical absorbance capacity (ORAC) values (particularly in *Aster scaber*, *Ligularia fischeri*, and *Cirsium setidens*) confirmed their superior ability to protect against oxidative damage through peroxyl radical inhibition. In an in vitro analysis, 3T3-L1 preadipocytes treated with 100 µg/mL of the extracts showed no cytotoxicity. The extracts also reduced intracellular ROS levels and lipid accumulation, with the exception of *Kalopanax pictus* extract. *Aster scaber* and *Ligularia fischeri* showed the highest potential among the selected crops; the other herbs also demonstrated notable activity [38] (Table 1).

Animal studies have demonstrated the antidiabetic potential of *C. lanceolata.* Jeong et al. investigated the effects of *C. lanceolata* water extract (CLW) on insulin sensitivity in non-obese rats with experimentally induced type 2 diabetes mellitus. Rats (partial pancreatectomized and fed a high-fat diet) were administered diets supplemented with either 0.3% CLW (CLW-L) or 1% CLW (CLW-H) for eight weeks. CLW-H significantly lowered serum glucose levels and urinary glucose loss compared with untreated controls and increased epididymal fat mass. During hyperinsulinemic–euglycemic clamp studies, CLW-treated rats exhibited higher glucose infusion rates (during hyperinsulinemic–euglycemic clamp) and lower hepatic glucose output under both basal and insulin-stimulated conditions than controls. At the molecular level, CLW improved hepatic insulin signaling, as indicated by increased phosphorylation of protein kinase B (Akt) and glycogen synthase kinase-3β (GSK-3β) in liver lysates (Table 1). These findings suggest that CLW enhances hepatic insulin sensitivity and improves insulin signaling in type 2 diabetic rats [39].

Insulin resistance induced by a high-fat/high-sucrose (HFHS) diet has been implicated in the development of various metabolic diseases, including nonalcoholic fatty liver disease (NAFLD) and diabetes mellitus [40,41]. Zhang et al. investigated the effects of *C. lanceolata* polysaccharide (CLPS) in an HFHS diet-induced insulin-resistant mouse model. Eight weeks of oral CLPS administration significantly suppressed the increase in blood glucose levels following glucose administration (glucose tolerance test, GTT) compared to that in the HFHS-only group. A decreased area under the curve (AUC) over 120 min further indicated improved insulin sensitivity, which was supported by the insulin tolerance test (ITT). In addition, fasting blood glucose (FBG), fasting insulin (FINS), and the insulin resistance index (homeostatic model assessment of insulin resistance [HOMA-IR]) were all lower in the CLPS-treated group than in the HFHS group [21] (Table 1). Insulin signaling is particularly vulnerable to impairment under redox-imbalanced conditions. ROS can impair insulin signaling by inducing abnormal serine/threonine phosphorylation of both the insulin receptor (IR) and insulin receptor substrate (IRS), disrupting the redistribution and recruitment of insulin signaling components, and reducing glucose transporter type (GLUT) translocation to the plasma membrane [42,43,44]. In the hepatic tissue, HFHS feeding decreased Akt phosphorylation at Ser473 and increased IRS-1 phosphorylation at Ser307, indicating impaired insulin signaling. In contrast, CLPS administration significantly increased phosphorylated (p)-Akt levels and reduced p-IRS-1 levels, suggesting its potential antidiabetic effects. To further examine the role of OS in HFHS-induced insulin resistance, hepatic antioxidant and redox parameters were assessed. Superoxide dismutase (SOD) and catalase (CAT) activity were reduced in the HFHS group, whereas CLPS attenuated these effects, significantly increasing SOD activity and restoring CAT activity. Malondialdehyde (MDA), a marker of lipid peroxidation [45], was reduced in CLPS-treated mice compared to mice fed HFHS alone. The glutathione:glutathione disulfide (GSH:GSSG) ratio, an index of the cellular redox status, was also increased in CLPS-treated mice. Moreover, nuclear factor erythroid 2-related factor 2 (Nrf2), a key transcriptional regulator of the cellular antioxidant response [46], which controls downstream effectors such as heme oxygenase-1 (HO-1), CAT, SOD, glutathione peroxidase (GPx), and NAD(P)H:quinone oxidoreductase 1 (NQO1) [47,48], was decreased in both the nuclear and cytoplasmic fractions of HFHS-fed mice. CLPS treatment upregulated Nrf2 expression in both compartments and increased the mRNA levels of HO-1 and NQO1, suggesting its potential antioxidant effects. Conversely, Kelch-like ECH-associated protein 1 (Keap1) was elevated in HFHS-fed mice but downregulated by CLPS, suggesting that CLPS may facilitate Nrf2 translocation [21] (Table 1).

### 4.2. Skeletal Muscle Atrophy

Skeletal muscle atrophy refers to the loss or reduction in muscle mass and strength resulting from an imbalance between protein synthesis and degradation. It can occur because of various conditions such as aging (sarcopenia), prolonged immobilization, severe burns, denervation, chronic diseases, malnutrition, or systemic inflammation [49]. When muscle atrophy coexists with excess adiposity, it contributes to the development of sarcopenic obesity [50], a condition in which chronic inflammation, insulin resistance, and lipid infiltration further accelerate muscle degradation while diminished muscle mass lowers metabolic rate and mobility, promoting additional fat accumulation [51]. This bidirectional interaction establishes a self-perpetuating cycle that exacerbates metabolic dysfunction and functional decline, thus highlighting the importance of understanding the mechanistic link between muscle atrophy and sarcopenic obesity. Because skeletal muscles play a vital role in metabolism, mobility, and overall health, preventing or reversing atrophy has become a major therapeutic target in pharmacological research.

To explore the potential of *C. lanceolata* to prevent muscle loss associated with obesity, its effects were evaluated in an in vivo model of sarcopenic obesity. Han and team investigated the effects of *C. lanceolata* in a high-fat diet (HFD)-induced sarcopenic obesity model of C57BL/6 mice [52]. Six-weeks of *C. lanceolata* to HFD-fed mice reduced body weight, epididymal, mesenteric, and perirenal fat, and adipocyte cross-sectional area. It also improved insulin signaling by reducing glucose, insulin, HOMA-IR, and intramyocellular triglyceride (TG) levels. Grip strength, muscle mass (quadriceps, gastrocnemius, and soleus muscles), and muscle fiber cross-sectional area increased after *C. lanceolata* administration. The phosphoinositide 3-kinase (PI3K)/Akt signaling, involved in regulating the synthesis and degradation of skeletal muscle protein, was also found to be activated as indicated by increased expression of p-mammalian target of rapamycin complex 1 (mTORc1)/total (t)-mTORc1, p-Akt/t-Akt, p-eukaryotic translation initiation factor 4E-binding protein 1 (4E-BP1)/t-4E-BP1, p-ribosomal protein S6 kinase beta-1 (S6K1)/t-S6K1, and p-forkhead box protein O3a (FoxO3a)/t-FoxO3a, and reduced expression of muscle RING-finger protein-1 (MuRF1) and Atrogin-1. *C. lanceolata* also improved skeletal muscle lipid metabolism, as evidenced by decreased expression of sterol regulatory element-binding protein 1c (SREBP-1c), diacylglycerol O-acyltransferase 2 (DGAT2) and stearoyl-CoA desaturase (SCD)1, together with increased expression of carnitine palmitoyl transferase (CPT)1, mitochondrial uncoupling protein (UCP)3, and peroxisomal acyl-coenzyme A oxidase (ACOX)1. In addition, serum total cholesterol (TC) and low-density lipoprotein (LDL) levels decreased, whereas high-density lipoprotein (HDL) levels and HDL/TC increased. High-performance liquid chromatography (HPLC) analysis identified tangshenoside I as the major bioactive constituent of the *C. lanceolata* water extract. We evaluated its effects on palmitic acid-induced muscle atrophy in C2C12 cells. This compound reversed the palmitic acid-induced decrease in myotube diameter and the increase in lipid accumulation (Table 1).

*C. lanceolata* and its bioactive compound, tangshenoside I, were further evaluated in both in vitro and in vivo models of muscle atrophy, including dexamethasone (Dex)-induced C2C12 myotubes and immobilization-induced C57BL/6 mice. Treatment with *C. lanceolata* and its bioactive compound tangshenoside I reversed the Dex-induced reduction in myotube diameter in C2C12 myotubes. Oral administration of *C. lanceolata* and tangshenoside I improved grip strength; increased the mass of the quadriceps, gastrocnemius, and soleus muscles; and restored the cross-sectional area (CSA) of gastrocnemius muscle fibers in immobilization-induced C57BL/6 mice. Muscle fiber CSA distribution shifted toward normal levels in both the *C. lanceolata*- and tangshenoside I-treated groups. Decreases in p-PI3K/t-PI3K, p-Akt/t-Akt, p-mTORC1/t-mTORC1, p-70 kDa ribosomal protein S6 kinase (p70S6K)/p70S6K, and p-4E-BP1/t-4E-BP1 levels were also reversed in *C. lanceolata* and tangshenoside I groups. Administration of *C. lanceolata* and tangshenoside I also increased p-FoxO3a/FoxO3a and decreased the FoxO3a sub-factors MuRF1 and Atrogin-1. The sirtuin 1 (SIRT1)/peroxisome proliferator-activated receptor gamma coactivator 1-alpha (PGC-1α) signaling is known to be involved in mitochondrial biogenesis [53], and was activated in *C. lanceolata* and tangshenoside I treatment groups. In addition, the mRNA expression levels of NRF1, NRF2, and mitochondrial transcription factor A (TFAM) were restored by treatment with *C. lanceolata* and tangshenoside I [26] (Table 1).

**Table 1:** In vitro and in vivo studies of *C. lanceolata* and bioactive compounds in diabetes, obesity and skeletal muscle atrophy.

Extract/Compound	Model	Treatment	Findings	Ref.
*C. lanceolata* methanolic extract (comparative)	Antioxidant assay	0.1–1 mg/mL	Antioxidant activity (DPPH and ABTS radical scavenging, FRAP, and reducing power activity).	[38]
	3T3-L1 cells	100 µg/mL (24 h, or every 2 days for 8 days)	↓ Lipid accumulation, ↓ ROS.	
*C. lanceolata* water extract	SD rats	HFD ± extract (0.3% or 1% diet) or rosiglitazone (20 mg/kg/day), 8 weeks (*n* = 16/group), OA via feed	↓ Serum glucose, ↓ AUC, ↑ insulin sensitivity, ↓ hepatic glucose output, preserved β-cell mass and function (↓ β-cell apoptosis), improved lipid metabolism (↓ TC, ↓ LDL, ↓ TG, ↑ HDL), ↑ CPT-1, ↑ SIRT1, ↑ glycogen, ↓ PEPCK, ↑ pAkt, ↑ pGSK-3β, ↑ pAMPK	[39]
*C. lanceolata* polysaccharide	C57BL/6 mice	Hight-fat high-sucrose diet ± polysaccharide (100 mg/kg), 8 weeks (*n* = 10/group), intragastric administration	↓ Blood glucose, ↓ AUC, ↓ serum insulin, ↓ HOMA-IR, ↑ p-Akt/Akt, ↓ p-IRS-1/IRS-1, ↑ SOD, ↑ CAT, ↓ MDA, ↑ GSH/GSSG, ↑ Nrf2, ↑ HO-1, ↑ NQO1, ↓ Keap1	[21]
*C. lanceolata* extract and Tangshenoside I	C57BL/6 mice	HFD ± extract (50, 100, or 200 mg/kg), 6 weeks (*n* = 8/group), OA via feed	↓ Body weight, ↓ adiposity, improved lipid metabolism (↓ TC, ↓ LDL, ↑ HDL/TC), ↑ grip strength, ↑ muscle mass, ↓ fasting blood glucose, ↓ insulin, ↓ HOMA-IR, ↓ intramyocellular triglyceride, ↑ p-Akt, ↑ p-mTORc1, ↑ p-4E-BP1, ↑ p-S6K1, ↑ p-FoxO3a, ↓ MuRF1, ↓ Atrogin-1, ↓ SREBP-1c, ↓ DGAT2, ↓ SCD1, ↑ CPT1, ↑ UCP3, and ↑ ACOX1	[52]
	C2C12 cells	PA (0.2 mM) ± Tangshenoside I (0.3 or 0.9 μg/mL), 24 h	↑ Myotube diameter, ↓ lipid accumulation	
*C. lanceolata* extract and Tangshenoside I	C2C12 cells	Dexa ± extract (0–50 µg/mL) or Tangshenoside I (0–1 µg/mL), 48 h	↑ Myotube diameter	[26]
	C57BL/6 mice	Immobilization ± extract (75, 150, and 300 mg/kg) or Tangshenoside I (0.5 mg/kg), 2 weeks (*n* = 7/group), OA	↑ Grip strength, ↑ muscle mass, ↑ p-PI3K, ↑ p-Akt, ↑ p-mTORc1, ↑ p-p70S6K, ↑ p-4E-BP1, ↑ p-FoxO3a, ↓ MuRF1, ↓ Atrogin-1, ↑ SIRT1, ↑ PGC-1α, ↑ Nrf1, ↑ Nrf2, ↑ TFAM	

**Abbreviations:** ↑, increase; ↓, decrease; DPPH, 2,2-diphenyl-1-picrylhydrazyl; ABTS, 2,2′-azino-bis (3-ethylbenzothiazoline-6-sulfonic acid); FRAP, ferric reducing antioxidant power; ROS, reactive oxygen species; HFD, high-fat diet; OA, oral administration; AUC, area under curve; TC, total cholesterol; LDL, low-density lipoprotein; TG, triglyceride; HDL, high-density lipoprotein; CPT-1, carnitine palmitoyltransferase I; SIRT1, sirtuin 1; PEPCK, phosphoenolpyruvate carboxykinase; p, phosphorylated; Akt, protein kinase B; GSK3β, glycogen synthase kinase-3 beta; AMPK, AMP-activated protein kinase; HOMA-IR, homeostatic model assessment for insulin resistance; IRS-1, insulin receptor substrate-1; SOD, superoxide dismutase; CAT, catalase; MDA, malondialdehyde; GSH/GSSG, reduced glutathione (GSH)/oxidized glutathione; Nrf2, nuclear factor erythroid 2-related factor 2; HO-1, heme oxygenase; NQO1, NAD(P)H:quinone oxidoreductase 1; Keap1, Kelch-like ECH-associated protein 1; mTORc1, mammalian target of rapamycin complex 1; 4E-BP1, eukaryotic translation initiation factor 4E-binding protein 1; S6K1, ribosomal protein S6 kinase beta 1; FoxO3a, forkhead box O3a; MuRF1, muscle RING finger-1; SREBP-1c, sterol regulatory element-binding protein 1; DGAT2, diacylglycerol O-acyltransferase 2; SCD1, stearoyl-CoA desaturase 1; UCP3, mitochondrial uncoupling protein 3; ACOX1, peroxisomal acyl-coenzyme A oxidase 1; PI3K, phosphoinositide 3-kinase; p70S6K, p-70 kDa ribosomal protein S6 kinase; PGC-1α, peroxisome proliferator-activated receptor-gamma coactivator 1-alpha; and TFAM, mitochondrial transcription factor A.

### 4.3. Cancer

OS and chronic inflammation are well-established contributors to the development and progression of cancer. Excessive production of ROS leads to DNA damage, genomic instability, and activation of oncogenic signaling pathways, whereas persistent inflammatory responses create a tumor-promoting microenvironment through the release of cytokines, chemokines, and growth factors. Interestingly, modulating ROS levels, either increasing or decreasing, can be leveraged therapeutically; elevated ROS levels can selectively trigger cancer cell death, while reducing ROS levels can prevent oxidative DNA damage and tumor initiation. Together, these processes not only initiate malignant transformation but also facilitate tumor growth, angiogenesis, and metastasis. Consequently, the targeting of OS and inflammation has emerged as a promising strategy for cancer prevention and therapy.

Several in vitro studies have demonstrated that *C. lanceolata* exerts anti-cancer effects by modulating OS, apoptosis, and cell cycle progression. A previous study investigated the anti-cancer activity of β-D-xylopyranosyl-(1→3)-β-D-glucuronopyranosyl echinocystic acid (codonoposide 1c), a triterpenoid saponin isolated from the roots of *C. lanceolata*, in HL-60 human promyelocytic leukemia cells. Codonoposide 1c exhibited marked cytotoxicity and induced apoptotic cell death as evidenced by DNA fragmentation, phosphatidylserine externalization, and activation of caspase-8, caspase-9, and caspase-3. The compound also promoted mitochondrial apoptotic signaling, as reflected by BH3-interacting domain death agonist (Bid) cleavage, cytochrome c release, second mitochondria-derived activator of caspase/direct IAP-binding protein with low pI (Smac/DIABLO) release, and reduced X-linked inhibitor of apoptosis protein (XIAP) expression. These findings suggest that codonoposide 1c exerts anti-cancer effects through the activation of mitochondrial and caspase-dependent apoptotic pathways [8]. The n-butanol fraction of *C. lanceolata* inhibited HT-29 human colon cancer cell growth in dose- and time-dependent manner. These effects were accompanied by an increase in the proportion of cells in the sub-G1 phase and elevated intracellular ROS levels. Polyamines such as putrescine, spermidine, and spermine are low-molecular-weight aliphatic amines involved in DNA stabilization, RNA and protein synthesis, ion channel regulation, membrane stability, and cellular growth. They are also important contributors to tumorigenesis. Treatment with the n-butanol fraction of *C. lanceolata* decreased cellular polyamine levels (spermidine and spermine) in HT-29 cells, suggesting the potential anti-cancer effects of *C. lanceolata*. The Bcl-2-associated X protein (Bax)/B-cell lymphoma 2 (Bcl-2) ratio, which serves as an indicator of apoptotic regulation [54], was enhanced upon treatment, along with increased expression of pro-apoptotic markers such as caspase-3, p53, and c-Jun N-terminal kinase (JNK). OS also downregulates the levels of survivin, an anti-apoptotic protein that functions as a caspase inhibitor [55]. The *C. lanceolata* fraction was also found to downregulate survivin expression, suggesting a potential mechanism underlying its anti-cancer activity [56] (Table 2).

Methanolic extracts of *C. lanceolata* and *Tricholoma matsutake* exhibit anti-cancer activity in HSC-2 human oral cancer cells. The extracts inhibited cell growth and induced apoptosis, as evidenced by nuclear condensation, nuclear fragmentation, and poly (ADP-ribose) polymerase (PARP) cleavage. The pro-apoptotic marker Bax was found to be upregulated in *C. lanceolata* exposed cells, while the Bcl-2 homologous antagonist/killer (Bak) protein (pro-apoptotic protein) was found to be enhanced in both extracts and suggested to be a key target molecule behind *C. lanceolata*-and *T. matsutake* mediated anti-cancer effects [57] (Table 2). Boo et al. evaluated the ethanolic extracts of *C. lanceolata* prepared at different ethanol concentrations. The 70% and 100% ethanolic extracts contained higher levels of TPC and TFC and exhibited dose-dependent DPPH radical scavenging activity, with the strongest activity observed in the 100% extract. These extracts also showed cytotoxic effects in several human cancer cell lines. The MCF-7 cells were relatively most affected with an IC_50_ value at 538.39 µg/mL, followed by HeLa and Calu-6 cells with IC_50_ of 637.87 µg/mL and 728.64 µg/mL in 70% ethanolic extract. In addition, the 70% extract at 800 and 1000 µg/mL exhibited pronounced cytotoxic effects on 3T3-L1 cells [58] (Table 2). Storage temperature and duration also influenced the bioactive properties of *C. lanceolata* root extracts. A 30% ethanolic extract stored at lower temperatures for shorter periods promoted T- and B-cell growth in a concentration-dependent manner, and exhibited higher reducing power and xanthine oxidase (XO) inhibitory activity. Furthermore, the cytotoxic activity against HeLa, Calu-6, and MCF-7 cells increased as storage temperature decreased from 45 °C to 5 °C [20] (Table 2).

Matrix metalloproteinases (MMPs), particularly MMP-2 and MMP-9, are key mediators of tumor progression because of their ability to degrade type IV collagen in the basement membrane, thereby promoting cancer cell invasion, metastasis, and angiogenesis [59]. Interestingly, the most abundant saponin in *C. lanceolata*, lancemaside A, was extracted from its roots, and its anti-invasive activity against ovarian cancer was investigated. In A2780 and SKOV3 human ovarian cancer cells, lancemaside A suppressed the migration, invasion, and expression of MMP-2 and MMP-9. Mechanistically, treatment with lancemaside A increased intracellular ROS levels, leading to the activation of the p38 mitogen-activated protein kinase (MAPK) pathway, thereby suggesting a potential anti-metastatic effect through the suppression of MMP expression and reduced cancer cell invasiveness [60] (Table 2).

**Table 2:** In vitro and in vivo studies of *C. lanceolata* and bioactive compounds in cancer.

Compound/Extract	Model	Treatment	Findings	Ref.
Codonoposide 1c (β-D-xylopyranosyl-(1 → 3)-β-D-glucuronopyranosyl echinocystic acid)	HL-60 cells	20–40 μM, 24 h	↑ Apoptosis, ↑ DNA fragmentation, ↑ caspase-8/-9/-3 activation, ↑ Bid cleavage, ↑ cytochrome c release, ↑ Smac/DIABLO release, ↓ XIAP	[8]
*C. lanceolata* n-butanol fraction	HT-29 cells	0–200 µg/mL, 24 or 48 h	↑ Cytotoxicity and apoptosis (sub-G1 arrest, ↑ Bax/Bcl-2, ↑ caspase-3, ↑ p53, ↑ JNK, ↓ survivin), ↑ ROS, ↓ polyamines	[56]
*C. lanceolata* methanolic extract (comparative)	HSC-2 cells	300–900 µg/mL, 24 or 48 h	↓ Cell growth and apoptosis (↓ PARP, ↑ DAPI-stained apoptotic cells, ↑ Bak, ↑ Bax)	[57]
*C. lanceolata* ethanolic extracts (30, 50, 70, and 100%)	Antioxidant assay	1–20 mg/mL	Total polyphenol content: 70 > 100 > 50 > 30; Total flavonoid content and DPPH activity: 100 > 70 > 50 > 30%	[58]
	HeLa, Calu-6, and MCF-7 cells	50–1000 µg/mL, 48 h	↑ Cytotoxicity (50 and 70—strongest); MCF-7 cells were the most sensitive.	
	3T3-L1 cells	Similar treatment as above	↓ Cell survival (70% strongest)	
*C. lanceolata* ethanolic extract (30%)	Antioxidant assay	0.5–5 mg/mL	↑ XO inhibitory activity and reducing power at low temp. and short period	[20]
	Human T and B cells	Extract stored at 5–45 °C for 0–90 days; 0.5 µg/mL, 10 days	↑ T and B cell number at low temp. and short period	
	HeLa, Calu-6 and MCF-7 cells	50–800 μg/mL, 48 h	↑ Cytotoxicity (MCF-7 were more prone)	
Lancemaside A	A2780 and SKOV3 cells	0–10 µM, A2780 cells, and 0–20 µM, SKOV3 cells; 0–48 h, and 0–60 min	↓ Migration, ↓ invasion, ↓ MMP-2, ↓ MMP-9, ↑ ROS, ↑ p-p38, ↑ p-JNK, ↑ p-ERK1/2	[60]

**Abbreviations:** ↑, increase; ↓, decrease; Bid, BH3-interacting domain death agonist; Smac/DIABLO, second mitochondria-derived activator of caspases/direct IAP-binding protein with low pI; XIAP, X-linked inhibitor of apoptosis protein; Bax, Bcl-2 associated X protein; Bcl-2, B-cell lymphoma; JNK, c-Jun N-terminal kinase; ROS, reactive oxygen species; PARP, poly(ADP-ribose) polymerase; Bak, Bcl-2 homologous antagonist/killer; DPPH, 2,2-diphenyl-1-picrylhydrazyl; XO, xanthine oxidase; MMP, matrix metalloproteinase; p, phosphorylated; and Erk1/2, extracellular signal-regulated kinases 1 and 2.

### 4.4. Hypogalactia

Postpartum hypogalactia is a term used to define a condition characterized by inadequate or absent milk secretion, and its prevalence is increasing [61]. Chen et al. observed that *C. lanceolata* polysaccharides attenuated HFD-induced postpartum hypogalactia in C57BL/6 mice. Oral administration of *C. lanceolata* polysaccharides reduced HFD-induced weight gain, hepatic steatosis, mesenteric adipocyte hypertrophy, and abnormal glucose/lipid metabolism. Mammary gland development, lactogenesis, and histological alterations also improved following the administration of *C. lanceolata* polysaccharides. Prolactin interacts with the prolactin receptor (PRLR) and activates Janus kinase (JAK)2/signal transducer and activator of transcription (STAT)5 signaling, which plays a critical role in lactogenesis and milk secretion [62]. The expression of prolactin and its receptor PRLR was increased in the mammary gland, and JAK2/STAT5 signaling was activated in *C. lanceolata* polysaccharide-treated group, suggesting a potentially beneficial role of *C. lanceolata* in experimental hypogalactia [22] (Table 3).

### 4.5. Cardiovascular Diseases

Vascular OS, characterized by an imbalance between ROS production and antioxidant defenses in blood vessels, plays a central role in the pathogenesis of cardiovascular diseases [63]. Excess ROS derived from sources such as nicotinamide adenine dinucleotide phosphate (NADPH) oxidases, mitochondria, XO, and uncoupled endothelial nitric oxide synthase (eNOS) induce endothelial dysfunction by reducing nitric oxide (NO) bioavailability, promoting inflammation, and enhancing vascular smooth muscle contraction [63]. These changes contribute to key pathological processes including atherosclerosis, hypertension, vascular remodeling, and thrombosis. OS accelerates plaque formation and vascular injury by oxidizing lipids, proteins, and DNA, ultimately increasing the risk of myocardial infarction, stroke, and heart failure [64]. Targeting vascular OS using antioxidants, enzyme modulators, and lifestyle interventions has emerged as a promising strategy for the prevention and management of cardiovascular diseases. Evidence from preclinical and clinical studies suggests that *C. lanceolata* may confer cardiovascular benefits by attenuating vascular OS, inflammation, and endothelial dysfunction.

A study performed by Han et al. reported the antihypertensive properties of *C. lanceolata* in an in vivo hypertension model generated by chronic immobilization and nicotine administration in rats. Treatment with *C. lanceolata* extract (200 and 400 mg/kg) reduced systolic blood pressure (SBP) and increased acetylcholine (ACh)- and sodium nitroprusside (SNP)-induced vascular relaxation in hypertensive rats. In addition, the anti-inflammatory properties of *C. lanceolata* were highlighted by decreased neutrophil counts after the administration of 200 mg/kg extract in hypertensive rats [23] (Table 3). Shin et al. demonstrated the cardioprotective effects of lancemaside A in reducing hypertension-induced SBP in a dose-dependent manner and the aortic expression of nicotinamide adenine dinucleotide phosphate oxidase (NOX)2 at doses of 20 and 40 mg/kg in hypertensive rats. Lancemaside A reduced the serum MDA levels. In addition, serum nitrite (NO^−^_2_) levels were higher in lancemaside A-treated rats, whereas eNOS induction in rat aortas was reduced at 20 and 40 mg/kg lancemaside A doses. Lancemaside A also reduced the aortic levels of nuclear factor kappa-light-chain-enhancer of activated B cells (NF-κB) and MAPKs, suggesting a potential anti-inflammatory role [65] (Table 3). The antihypertensive effects of *C. lanceolata* extract were further demonstrated in a double-blind randomized controlled human clinical trial in prehypertensive individuals. A 6-week administration of *C. lanceolata* extract to 80 individuals aged 19–60 years with elevated blood pressure (systolic BP, 120–139 mmHg; diastolic BP 80–89 mmHg) reduced SBP and increased CAT activity. Serum NO^−^_2_ concentration also increased compared with baseline in *C. lanceolata* administered elevated SBP subgroup. Among individuals with stage 1 hypertension, C-reactive protein (CRP) and MDA levels were reduced in the *C. lanceolata* -given group compared to the placebo group. Notably, while the atherogenic LDL/HDL ratio increased in both groups over the 6-week intervention period, the increase was significantly greater in the placebo group. Although the LDL/HDL ratio did not improve compared with baseline, the smaller increase observed in the *C. lanceolata*-treated group suggests a potential mitigating effect on the progression of unfavorable lipid profile changes associated with prehypertension. These findings, together with the observed reduction in inflammatory and OS markers, support the possible beneficial role of *C. lanceolata* extract in cardiovascular health [66] (Table 3). Kim et al. investigated the effects of *C. lanceolata* extract on the vascular tension in rat carotid arteries using myography. The extract increased vasorelaxation in endothelium-impaired arteries exposed to either normal or high extracellular potassium concentrations [K^+^]_o_. Also, the extract inhibited Ca^2+^ induced vasoconstriction through voltage-dependent Ca^2+^ channels (VDCCs) [67] (Table 3).

### 4.6. Liver Injury

Inflammation and OS are closely interconnected processes that play pivotal roles in the development and progression of liver disease. Excessive production of ROS arising from the mitochondria, cytochrome P450 enzymes, or inflammatory cells overwhelms the antioxidant defense system, leading to lipid peroxidation, protein modification, and DNA damage in hepatocytes. This oxidative injury activates Kupffer cells and hepatic stellate cells, promoting the release of pro-inflammatory cytokines such as tumor necrosis factor (TNF-α), interleukin (IL)-1β, and IL-6, which further amplify ROS generation and create a self-perpetuating cycle of damage. The combined effects of inflammation and OS contribute to hepatocellular apoptosis, necrosis, and fibrogenesis, ultimately driving the progression of liver disorders including NAFLD, alcoholic liver disease, hepatitis, and cirrhosis. Kim et al. reported the protective effects of the saponin fraction of *C. lanceolata* against stress-induced liver damage by reducing serum glutamic-oxaloacetic transaminase (GOT) and glutamic–pyruvic transaminase (GPT) levels in an ICR mouse model and found that it reduced SNP-induced ROS generation, inhibited NO production, and enhanced radical scavenger activity in RAW264.7 [25] (Table 3).

Cho et al. investigated the protective effects of *C. lanceolata* root water extract against alcoholic fatty liver in SD rats chronically fed an ethanol-containing diet for eight weeks. Supplementation with *C. lanceolata* significantly attenuated ethanol-induced hepatic triglyceride and cholesterol accumulation. Mechanistically, the extract suppressed the expression of inflammatory and lipogenic mediators, including TNF-α, liver X receptor alpha (LXRα), sterol regulatory element-binding protein-1c (SREBP-1c), 3-hydroxy-3-methylglutaryl-coenzyme A reductase (HMGR), and low-density lipoprotein receptor (LDLR), while restoring AMP-activated protein kinase (AMPK) and acetyl-CoA carboxylase (ACC) phosphorylation. These findings suggest that *C. lanceolata* protects against alcoholic fatty liver through the coordinated regulation of inflammation, lipid metabolism, and AMPK-associated energy homeostasis [6]. Cha et al. investigated the antilipogenic and anti-inflammatory effects of the root extract of *C. lanceolata* in vivo using a chronic ethanol-fed mouse model. Ethanol was provided as a liquid diet (5% *w*/*v*, 36% energy) for nine weeks. Among the three different root fractions prepared, the methanol extract (CME) exhibited the most marked attenuation of alcohol-induced hepatic steatosis, as shown by reductions in hepatic free fatty acid concentration, body weight loss, and hepatic accumulation of TG and cholesterol. CME significantly restored ethanol-induced downregulation of the adiponectin receptors (adipoR1 and adipoR2). It also modulated downstream molecules involved in fatty acid oxidation, including peroxisome proliferator-activated receptor α (PPARα), carnitine palmitoyltransferase 1 (CPT1), mitochondrial trifunctional protein (MTP), long-chain acyl-CoA dehydrogenase (LCAD), and medium-chain acyl-CoA dehydrogenase (MCAD). Furthermore, CME regulated adipogenesis-associated factors, including PPARγ, retinoid X receptor (RXR), CCAAT/enhancer-binding protein alpha (C/EBPα), lipoprotein lipase (LPL), adipocyte protein 2 (aP2), and fatty acid synthase (FAS), indicating suppression of ethanol-induced lipogenic responses. Additionally, CME markedly reversed ethanol-induced activation of toll-like receptor (TLR)-mediated inflammatory signaling pathways. This was accompanied by reduced expression of lipopolysaccharide-binding protein (LBP), cluster of differentiation (CD)14, myeloid differentiation primary response 88 (MyD88), MyD88 adapter-like (MAL), toll-interleukin-1 receptor (TIR) domain-containing adaptor-inducing interferon-β (TRIF), TNF receptor-associated factor (TRAF)6, and several interferon regulatory factor (IRF) family members. CME also decreased NF-κB p50/p65 activation and reduced the expression of downstream inflammatory mediators, including TNF-α, IL-6, interferon (IFN)-α, IFN-β, IL-12p40, and chemokine (C-X-C motif) ligand 2 (CXCL2). The authors proposed that the beneficial effects of CME against alcoholic fatty liver may be mediated through adenosine- and adiponectin-associated regulation of hepatic steatosis, together with the modulation of TLR-mediated inflammatory signaling pathways [68] (Table 3).

**Table 3:** In vitro and in vivo studies of *C. lanceolata* and bioactive compounds in hypogalactia, hypertension and liver injury.

Compound/Extract	Model	Treatment	Findings	Ref.
*C. lanceolata* polysaccharide	C57BL/6 mice	HFD ± polysaccharide (300 mg/kg), 8 weeks (*n* = 20/group); OA, evaluated from puberty through lactation	↓ Body weight gain, ↓ liver weight, ↓ mesenteric adipose tissue, ↓ hepatic vacuolation, ↓ glucose, ↓ non-esterified fatty acid, ↓ TC, ↓ TG, ↑ lactation performance (↑ milk yield, lactose, triglycerides, and casein), ↑ offspring growth and survival, improved mammary gland development and differentiation, ↑ GATA-3, ↑ PRL, ↑ PRLR, ↑ p-JAK2, ↑ Nrg1, ↑ p63, ↑ ERBB4, and ↑ p-STAT5	[22]
*C. lanceolata* extract	SD rats	Normotensive ± extract; hypertensive ± extract (200 and 400 mg/kg) or PC (nifedipine, 10 mg/kg), 3 weeks	↓ SBP, ↑ vascular relaxation, ↓ neutrophil in hypertensive rats	[23]
Lancemaside A	SD rats	Hypertensive ± lancemaside A (1, 20, and 40 mg/kg) or PC (nifedipine, 10 mg/kg), *n* = 8–9/group, OA	↓ SBP, ↓ aortic NOX2, ↓ serum MDA, ↓ aortic eNOS, ↑ serum nitrite, ↓ endothelium thickness, ↓ aortic NF-κB, and ↓ MAPK signaling (↓ p-p38, ↓ p-JNK, ↓ p-ERK)	[65]
*C. lanceolata* extract	Double-blind, RCT	Prehypertensive and stage 1 hypertensive adults; placebo or extract (1000 mg/day), 2 capsules daily, 6 weeks (*n* = 80)	↓ SBP, ↓ DBP (stage 1 hypertensive group), ↑ CAT, ↑ serum nitrite, ↓ hs-CRP, ↓ MDA, ↑ LDL/HDL (change from baseline)	[66]
*C. lanceolata* extract	SD rats	Isolated rat carotid arteries; extract (50 and 250 µg/mL) in vasoconstriction and vasorelaxation experiments, OA via diet	↑ Vasorelaxation and↓ vasoconstriction, including in endothelium-impaired arteries, partly through inhibition of voltage-dependent Ca^2+^ channels	[67]
*C. lanceolata* saponin fraction	ICR mice	Forced-swimming stress model; saponin fraction (50 mg/kg) or vitamin C (500 mg/kg), OA	↓ Serum GOT, ↓ serum GPT	[25]
	RAW264.7 cells	Saponin fraction (100 or 200 µg/mL) prior to SNP (250 µM) or LPS (1 µg/mL) stimulation	↓ ROS, ↓ NO, ↑ cell viability, ↓ generation of radicals	
*C. lanceolata* root extract	SD rats	Alcohol-fed rats ± root extract (0.5%), 8 weeks	↓ Hepatic TG and cholesterol; ↓ TNF-α, ↓ LXRα, ↓ SREBP-1c, ↓ HMGR, ↓ LDLR; ↑ p-AMPK, ↑ p-ACC; attenuated alcoholic fatty liver	[6]
*C. lanceolata* extract	C57BL/6N mice	Ethanol-fed mice ± methanol extract, ethanol supernatant, or ethanol precipitate, 9 weeks (*n* = 8/group), OA via diet	↑ Body weight, ↓ serum FFA,↓ ALT & ↓ AST, ↓ hepatic lipid accumulation and liver enlargement,↓ hepatic TG, ↓ cholesterol, & ↓ FFA, ↓ adenosine receptors (A1R, A2AR, A2BR, A3R), improved fatty acid oxidation (↑ PPARα, ↑ CPT-1, ↑ MTP, ↑ LCAD, ↑ MCAD), suppressed lipogenesis (↓ PPARγ, ↓ RXR, ↓ C/EBPα, ↓ LPL, ↓ aP2, ↓ FAS, ↓ SREBP-1c), ↑ adipoR1, ↑ adipoR2, ↑ SIRT1, ↑ PGC1α, ↑ p-AMPK, ↑ p-ACC, and suppressed TLR-mediated inflammatory signaling (↓ TLRs, ↓ LBP, ↓ CD14, ↓ MyD88, ↓ MAL, ↓ TRIF, ↓ TRAF6, ↓ IRFs, ↓ p50/p65, ↓ TNF-α, ↓ IL-6, ↓ IFN-α, ↓ IFN-β, ↓ IL-12p40, ↓ CXCL2).	[68]

**Abbreviations:** ↑, increase; ↓, decrease; HFD, high-fat diet; OA, oral administration; TC, total cholesterol; TG, triglyceride; GATA-3, GATA binding protein 3; PRL, prolactin; PRLR, prolactin receptor; p, phosphorylated; JAK2, Janus kinase 2; Nrg1, neuroregulin1; ERBB4, receptor protein-tyrosine kinase ERBB-4; Stat-5, signal transducers and activators of transcription 5; PC, positive control; SBP, systolic blood pressure; NOX2, nicotinamide adenine dinucleotide phosphate oxidase; MDA, malondialdehyde; eNOS, endothelial nitric oxide synthase; NF-κB, nuclear factor kappa B; MAPK, mitogen-activated protein kinase; JNK, Jun N-terminal kinase; ERK, extracellular signal-regulated kinase; RCT, randomized controlled trial; DBP, diastolic blood pressure; CAT, catalase; hs-CRP, high-sensitivity C-reactive protein; LDL, low-density lipoprotein; HDL, high-density lipoprotein; SNP, sodium nitroprusside; LPS, lipopolysaccharide; GOT, glutamate–oxalacetate transaminase; GPT, glutamate–pyruvate transaminase; ROS, reactive oxygen species; NO, nitric oxide; TNF-α, tumor necrosis factor-alpha; LXRα, liver X receptor-alpha; HMGR, 3-hydroxy-3-methylglutaryl-coenzyme A reductase; LDLR, low-density lipoprotein receptor; FFA, free fatty acid; ALT, alanine aminotransferase; AST, aspartate aminotransferase; A1R/A2R/A2BR/A3R, adenosine receptors; PPAR, peroxisome proliferator-activated receptor; CPT-1, carnitine palmitoyltransferase; MTP, microsomal triglyceride transfer protein; LCAD, long-chain acyl-CoA dehydrogenase; MCAD, medium-chain acyl-CoA dehydrogenase; RXR, retinoid X receptor; C/EBPα, CCAAT/enhancer-binding protein alpha; LPL, lipoprotein lipase; aP2, adipocyte protein 2; FAS, fatty acid synthase; SREBP-1c, sterol regulatory element binding transcription factor 1; adipoR1/2, adiponectin receptor 1/2; SIRT1, silent-mating-type information regulation 2 homolog 1; PGC1α, peroxisome proliferative activated receptor gamma coactivator 1 alpha; AMPK, AMP-activated protein kinase; ACC, acetyl-CoA carboxylase; TLR, toll like receptor; LBP, LPS binding protein; CD, cluster of differentiation; MyD88, myeloid differentiation primary response 88; MAL, myelin and lymphocyte protein; TRIF, TLR adaptor molecule; TRAF6, TNF-receptor-associated factor 6; IRF, interferon regulatory factors; IL, interleukin; IFN, interferon; and CXCL2, chemokine (C-X-C motif) ligand 2.

### 4.7. Neurodegenerative Disorders

ACh is an important neurotransmitter ubiquitously present in the nervous system and is synthesized by choline acetyltransferase (ChAT) in cholinergic neurons. ACh plays a key role in the regulation of learning, memory and behaviors [69]. In neuronal signaling, acetylcholinesterase (AChE) is an important enzyme responsible for the degradation of ACh, which in turn blocks postsynaptic signal transmission [70]. Cholinergic transmission impairment can cause cognitive decline linked to aging and dementia, and patients with Alzheimer’s disease (AD) have been reported to have lower levels of ACh in the brain owing to the loss of cholinergic neurons in the basal forebrain [71]. Thus, the central cholinergic system is an important target for the development of drugs to treat AD. AChE inhibitors have attracted the attention of researchers as the most promising drugs against AD because they can increase the availability of ACh in central cholinergic synapses [72].

Scopolamine (SCP) is a non-selective muscarinic ACh receptor antagonist known to impair short-term memory and learning acquisition [73]. Jung et al. evaluated the cognitive effects of lancemaside A, a triterpenoid saponin isolated from *C. lanceolata*, and its metabolite echinocystic acid, in vivo using SCP-induced memory-impaired ICR mice [74]. The study found that both lancemaside A and echinocystic acid significantly reversed SCP-induced deficits in behavioral tasks (passive avoidance, Y-maze, and Morris water maze) and inhibited AChE activity with IC_50_ values of 13.6 µM and 12.2 µM, respectively. In addition, both compounds increased the expression of brain-derived neurotrophic factor (BDNF), and phosphorylated cAMP response element-binding protein (CREB), which is known to be involved in cognitive enhancement [75]. These findings suggest a potential neuroprotective mechanism involving cholinergic modulation and neurotrophic signaling. The authors also proposed that orally administered lancemaside A may be metabolized to echinocystic acid (by intestinal microflora), which may exert memory-improving effects [74] (Table 4).

In a study by Weon et al., steamed and fermented *C. lanceolata* extract (SFCE) demonstrated neuroprotective effects in both in vivo and in vitro models, improving SCP-induced memory impairment in ICR mice and displaying cytoprotective effects in glutamate-exposed HT-22 neuronal cells. In behavioral tests, mice treated with SFCE (300 mg/kg) exhibited significantly reduced escape latencies in the Morris water maze and increased step-through latency in the passive avoidance test compared with SCP-only mice. In HT-22 cells exposed to glutamate, SFCE (500 µg/mL) provided dose-dependent neuroprotection against cytotoxicity. Chemical analysis revealed that steam-fermentation markedly increased the levels of phenolic acids in the extract and that gallic acid and vanillic acid were more abundant in the SFCE than in the non-processed extract. The authors suggested that these increases in antioxidant phenolics may underlie the cognition-enhancing and neuroprotective effects of SFCE [24] (Table 4). Another report by a similar research team demonstrated the cognition-enhancing potential of steamed and fermented *C. lanceolata* in mice with SCP-induced memory impairment. The fermented extract (administered at 300, 500, and 800 mg/kg) significantly improved the performance in the Morris water maze and passive avoidance tests in SCP-treated ICR mice. Biochemically, the treatment reduced AChE activity in the brain and markedly increased the hippocampal levels of p-CREB and BDNF compared with SCP alone [76] (Table 4). Weon et al. investigated the mechanism underlying the neuroprotective properties of steamed and fermented *C. lanceolata* in vitro using glutamate-induced toxicity in HT-22 cells. Steamed and fermented *C. lanceolata* reduced glutamate-induced cell death, inhibited morphological alterations, and increased the cell density. OS is considered a major contributor to the development of NDs as it damages lipids, proteins, and DNA in neuronal cells, leading to neuronal cell death [77,78]. Steamed and fermented *C. lanceolata* reduced intracellular ROS levels, restored the mitochondrial membrane potential, reduced Ca^2+^ influx and NO production, and prevented glutathione reductase depletion in glutamate-treated HT-22 cells. It also reduced the expression of apoptotic markers including Bax and caspase-3 [79] (Table 4).

The same research team reported the neuroprotective effects of steamed and fermented EtOH extract of *C. lanceolata* in vivo using an amyloid-beta (Aβ)-induced memory impairment model in ICR mice. The extract attenuated amyloid-beta (Aβ)-induced memory impairment, inhibited AChE activity, and activated CREB/BDNF/extracellular signal-regulated kinase (ERK) expression in ICR mice. Furthermore, ultra-performance liquid chromatography quadrupole time-of-flight mass spectrometry (UPLC-QTOF-MS) analysis identified lancemasides A, B, C, D, E, and G, as well as fetidissimoside A, in the extract [80] (Table 4). In another in vivo study, Yoo et al. reported the neuroprotective effects of raw and steamed ethyl acetate extracts of *C. lanceolata* in a gerbil model of transient cerebral ischemia. The treatment reduced reactive gliosis and ischemic neuronal loss while increasing SOD1 and BDNF expression [81] (Table 4).

### 4.8. Pulmonary Injury

Pulmonary inflammation and injury often occur in response to infections, toxins, or environmental pollutants, leading to disruption of the alveolar–capillary barrier, edema, and impaired gas exchange. Alveolar macrophages (AMs) are resident immune cells in the lung alveoli that act as the first line of defense and detect pathogens and damaged cells via pattern recognition receptors (PRRs) [82]. TLRs are PRRs present on innate immune cells, such as microglia, dendritic cells, macrophages, monocytes, neutrophils, mast cells, basophils, and natural killer cells [83], and are activated by specific pathogen-associated molecular patterns (PAMPs) present on microbes, triggering immune signaling pathways and initiating an inflammatory response [84].

Lipopolysaccharide (LPS), a potent ligand of TLR4, activates the receptor and triggers the MyD88-dependent pathway. The adaptor proteins TIR domain-containing adaptor protein (TIRAP) and MyD88 are recruited to initiate a signaling cascade. This cascade involves the kinases interleukin-1 receptor-associated kinase (IRAK)4, IRAK1, and TRAF6, ultimately leading to the activation of the transcription factor NF-κB [84]. When NF-κB is activated, it translocates from the cytoplasm to the nucleus and binds to κB enhancer elements of target genes, leading to the production of pro-inflammatory cytokines such as IL-1β, TNF-α, IL-6, and chemokines [85].

Joh et al. observed the anti-inflammatory activity of echinocystic acid in LPS-stimulated alveolar macrophages, both in vitro and in vivo [86]. Echinocystic acid was found to attenuate LPS-induced protein expression of pro-inflammatory mediators such as IL-1β, TNF-α, prostaglandin E2 (PGE2), NO^−^_2_, inducible nitric oxide synthase (iNOS), and cyclooxygenase-2 (COX-2) in alveolar macrophages. Phosphorylation of inhibitor of nuclear factor-kappa B kinase subunit beta (IKK-β), inhibitor of nuclear factor-kappa B alpha (IκB-α) degradation, and NF-κB phosphorylation were also decreased. Echinocystic acid also prevented the interaction between LPS and TLR4 and inhibited IRAK-1 degradation in alveolar macrophages. In vivo data showed decreased albumin protein, neutrophils, along with pro-inflammatory mediators such as TNF-α and IL-1β, in bronchoalveolar lavage fluid (BALF) after oral administration of echinocystic acid in LPS-induced mice. Treatment with echinocystic acid also inhibited myeloperoxidase activity. In addition, echinocystic acid reduced TNF-α and IL-1β levels and suppressed NF-κB activation in lung tissue, along with attenuating LPS-induced pulmonary edema [86] (Table 4).

Allergic asthma is a chronic inflammatory airway disease characterized by an increased sensitivity to environmental allergens. Exposure to these allergens activates T helper type 2 (Th2) cells, leading to the release of Th2 cytokines (IL-4, IL-5, and IL-13) that drive inflammation, mucus production, and airway hyperresponsiveness [87]. Seo et al. demonstrated the anti-inflammatory activity of *C. lanceolata* in in vivo a BALB/c mouse model of asthma. Oral administration of *C. lanceolata* decreased inflammatory cell infiltration into peribronchiolar and perivascular lesions, and inhibited mucus hypersecretion and goblet cell hyperplasia in lung tissues. *C. lanceolata* also reduced the number of inflammatory cells, especially eosinophils, and increased the number of alveolar macrophages in the BALF. Th2 related cytokines, including IL-4, IL-5, IL-6, and IL-13, which play important roles in allergic inflammation, were suppressed in *C. lanceolata* given group. In addition, *C. lanceolata* reduced Th2 cell differentiation and activation, induced the expression of SOD2, and activated Treg cells. Furthermore, *C. lanceolata* extract induced IL-10 production in BALF and LPS-stimulated RAW264.7 cells [7] (Table 4). These findings suggest that *C. lanceolata* has therapeutic potential against allergic lung inflammation by modulating Th2 responses and OS.

Recently, a study was conducted on Reliea^®^ (RelA), a combination made up of *C. lanceolata* and *Chaenomeles sinensis* extract and showed anti-inflammatory activity of the RelA against particulate matter diesel exhaust particles (PM_10_D)-induced in vitro and in vivo models of respiratory disease [88]. Lancemaside A and protocatechuic acid were identified as the major compounds in *C. lanceolata* and *C. sinensis*, respectively, by HPLC analysis. The RelA was found to reduce ROS and NO, along with the pro-inflammatory mediators such as IL-6, IL-1β, CXCL-2, TNF-α and monocyte chemotactic protein-1 (MCP-1) expression in PM_10_-induced MH-S cells. RelA also inhibited IRAK1 and the phosphorylation of NF-κB, MAPK/ERK, nucleotide-binding oligomerization domain (NOD)-, leucine-rich repeat (LRR)-, pyrin domain (PYD)-containing protein 3 (NLRP3), and caspase-1 expression. Symmetric dimethylarginine (SDMA), an endogenous nitric oxide synthase inhibitor implicated in OS, inflammation, and endothelial dysfunction [89], was also reduced in the serum of PM_10_D-exposed mice. Histopathological alterations such as inflammatory cell infiltration, airway wall thickening, fibrosis, and hyperplasia of goblet cells were ameliorated by RelA administration. RelA effectively suppressed PM_10_D-induced immune overactivation by reducing neutrophils and other inflammatory cells in BALF, lung tissue, and peripheral blood mononuclear cells (PBMCs). Consistent with these findings, RelA reduced BALF levels of CXCL-1, macrophage inflammatory protein (MIP)-2, IL-17, TNF-α, and IL-1α, indicating attenuation of airway inflammation. RelA also downregulated the expression of mucin 5AC (MUC5AC), thymus and activation-regulated chemokine (TARC), CXCL-1, transient receptor potential ankyrin 1 (TRPA1), transient receptor potential vanilloid 1 (TRPV1), and COX-2 in the lung tissues. In addition, the treatment reduced F4/80-positive macrophages and IL-1α-positive cells within pulmonary tissue. Moreover, RelA suppressed the activation of NF-κB and MAPK signaling pathways, as evidenced by reduced p-NF-κB, p-JNK, and p-ERK expression. Furthermore, RelA improved airway function and overall pulmonary health, partly through its expectorant effects [88] (Table 4).

**Table 4:** In vitro and in vivo studies of *C. lanceolata* and bioactive compounds in neurodegenerative disorders and pulmonary injury.

Compound/Extract	Model	Treatment	Findings	Ref.
Lancemaside A and echinocystic acid	AChE activity	Lancemaside A or echinocystic acid (0–50 µM); PC (donepezil)	↑ AChE inhibition	[74]
	ICR mice	SCP ± lancemaside A or echinocystic acid (10 and 20 mg/kg, p.o.) or donepezil or tacrine, n = 10/group, OA	↓ Memory and learning deficits (↑ step-through latency, ↓ escape latency, ↑ spontaneous alteration), ↑ BDNF, ↑ p-CREB	
*C. lanceolata* steamed and fermented extract	Steaming and fermentation	Steamed and probiotic-fermented extract	↑ Gallic acid, ↑ vanillic acid	[24]
	ICR mice	SCP ± steamed and fermented or non-fermented extract (100 and 300 mg/kg) or donepezil, n = 7/group, OA	↓ Escape latency, ↓ distance to platform, ↑ swimming time in target quadrant, ↑ step-through latency time	
	HT-22 cells	Glutamate ± steamed and fermented or non-fermented extract (10–500 µg/mL) or Trolox, 24 h	↑ Cell viability	
*C. lanceolata* steamed and fermented extract	Steaming and fermentation	Steamed and probiotic-fermented extract		[76]
	ICR mice	SCP ± steamed and fermented extract (300, 500, and 800 mg/kg) or donepezil, *n* = 7/group, OA	↓ Escape latency, ↑ swimming time in target quadrant, ↓ average distance to platform, ↑ latency time, ↑ AChE inhibition, ↑ BDNF, ↑ p-CREB	
*C. lanceolata* steamed and fermented extract	Steaming and fermentation	Steamed and probiotic-fermented extract	↑ Gallic acid, ↑ vanillic acid, ↑ trans-ferulic acid	[79]
	HT-22 cells	Glutamate ± steamed and fermented extract (10–500 µg/mL) or Trolox	↓ Cell death, ↓ morphological change,↓ ROS, ↑ mt-membrane potential,↓ Ca^2+^ concentration, ↓ NO, ↑ GSH, ↑ GR, ↓ Bax, ↓ caspase-3, ↑ DPPH radical scavenging activity	
*C. lanceolata* steamed and fermented extract	Steaming and fermentation	Steamed and probiotic-fermented extract	Presence of lancemasides A, B, C, D, E, and G, and foetidissimoside A	[80]
	ICR mice	Aβ peptide aggregate ± steamed and fermented extract (300, 500, and 800 mg/kg) or donepezil, *n* = 7/group, OA	↓ Escape latency, ↓ mean swimming distance, ↑ swimming time in target quadrant, ↑ latency time, ↑ AChE inhibition, ↑ BDNF, ↑ p-CREB, ↑ p-ERK	
*C. lanceolata* ethyl acetate extracts (raw and steamed)	Male Mongolian gerbils	Ischemic group ± raw and steamed extracts and solvent fractions (50 mg/kg), once daily, 1-week, *n* = 7/group, OA	↑ CV+ and NeuN+ neurons, ↓ F-J B+ neurons, ↓ IB4+ microglial activation, ↓ GFAP+ astrocyte activation, and ↑ SOD1 and BDNF immunoreactivity (ethyl acetate fractions were most effective)	[81]
Echinocystic acid	C57BL/6 mice	LPS-induced acute lung injury		[86]
	Mouse alveolar macrophages	LPS or PGN ± echinocystic acid (1–10 µM), 20 h	↓ IL-1β, ↓ TNF-α, ↓ PGE2, ↓ NO, ↓ iNOS, ↓ COX-2, ↓ p-IKKβ, ↑ IKB-α, ↓ p-p65, ↓ p-ERK, ↓ p-JNK, ↓ p-p38, ↓ LPS to alveolar macrophage binding, ↑ IRAK-1	
	C57BL/6 mice	LPS ± echinocystic acid (2.5 or 5 mg/kg) or dexamethasone, OA	↓ Albumin, ↓ neutrophils ↓ TNF-α, ↓ IL-1β, and ↓ MPO activity, ↓ TNF-α, ↓ IL-1β, ↓ NF-κB activation (lungs), ↓ lung edema	
*Codonopsis lanceolata* extract	Balb/C mice	Ovalbumin ± extract (200 and 400 mg/kg) or dexamethasone, 7 days (*n* = 7/group)	↓ Peribronchiolar and perivascular inflammatory infiltration, ↓ mucus hypersecretion, ↓ goblet cell hyperplasia, ↓ total inflammatory cells, ↓ eosinophils, ↑ macrophages, ↓ IL-4, ↓ IL-5, ↓ IL-6, ↓ total IgE, ↓ activated CD4^+^ T cells, ↑ IL-10, ↑ SOD2, ↑ FoxP3, ↓ Gata3	[7]
	CD4^+^ T cells (Th2 cells)	Th2-polarized CD4^+^ T cells ± extract	↓ IFNγ, ↓ IL-5, ↓ Gata3^+^ cell	
	RAW264.7 cells	LPS ± extract (200 or 400 µg/mL)	↑ IL-10	
Reliea^®^ (*C. lanceolata* and *Chaenomeles sinensis* combination), and Lancemaside A and protocatechuic acid	Alveolar macrophage MH-S cells	PM_10_ (50 µg/mL) ± *C. lanceolata* or *C. sinesis* extract (200 µg/mL) or Reliea^®^ (100 and 200 µg/mL) or lancemaside A (10 µg/mL) or protocatechuic acid (30 µg/mL), 18 h	↓ ROS, ↓ NO, ↓ IL-6, ↓ IL-1β, ↓ MCP-1, iNOS, ↓ CXCL-2, ↓ TNF-α, IL-6, ↓ IRAK1, ↓ p-NF-κB, ↓ p-NLRP3, ↓ caspase-1, ↓ p-ERK	[88]
	BALB/c mice	PM_10_D-sensitized control ± *C. lanceolata* or *C. sinesis* extract (200 mg/kg) or dexamethasone or Reliea^®^ (100, 200, and400 mg/kg) or lancemaside A (10 mg/kg) or protocatechuic acid (30 mg/kg), 12 days (*n* = 8/group), OA	↓ SDMA, ↓ lung and tracheal histopathological alterations ↓ BALF and lung inflammatory cells, ↓ CD4^+^, ↓ CD8^+^, ↓ neutrophil- and eosinophil-associated populations, ↓ CXCL-1, ↓ MIP2, ↓ IL-17, ↓ TNF-α, ↓ IL-1α, ↓ MUC5AC, ↓ TARC, ↓ TRPA1, ↓ TRPV1, ↓ COX-2, ↓ F4/80, ↓ p-NF-κB, ↓ p-JNK, ↓ p-ERK, ↑ phenol-red secretion, and improved airway and lung function	

**Abbreviations:** ↑, increase; ↓, decrease; AChE, acetylcholinesterase; PC, positive control; SCP, scopolamine; OA, oral administration; p, phosphorylated; BDNF, brain-derived neurotrophic factor; CREB, cAMP response element-binding protein; ROS, reactive oxygen species; NO, nitric oxide; GSH, glutathione; GR, glutathione reductase; Bax, Bcl-2 associated X; DPPH, 2,2-diphenyl-1-picrylhydrazyl; Aβ, amyloid beta; ERK, extracellular signal-regulated kinase; GFAP, glial fibrillary acidic protein; SOD, superoxide dismutase; LPS, lipopolysaccharides; PGN, peptidoglycan; IL, interleukin; TNF-α, tumor necrosis factor-alpha; PGE2, prostaglandin E2; iNOS, inducible nitric oxide synthase; COX-2, cyclooxygenase-2; IKKβ, inhibitor of nuclear factor kappa-B kinase subunit beta; IKB-α, inhibitor of kappa B-alpha; JNK, c-Jun N-terminal kinase; IRAK-1, interleukin-1 receptor-associated kinase 1; MPO, myeloperoxidase; NF-κB, nuclear factor kappa-light-chain-enhancer of activated B cells; FoxP3, forkhead box P3; GATA3, GATA binding protein 3; PM_10_D, particulate matter 10 plus diesel exhaust particles; MCP-1, monocyte chemoattractant protein-1; CXCL-2; chemokine (C-X-C motif) ligand 2; NLRP3, NLR family pyrin domain containing 3; SDMA, symmetric dimethyl-arginine; BALF, bronchoalveolar lavage fluid; MIP2, macrophage inflammatory protein-2; MUC5AC, mucin 5AC; TARC, thymus and activation-regulated chemokine; TRPA1, transient receptor potential ankyrin 1; TRPV1, transient receptor potential vanilloid 1.

### 4.9. Anti-Microbial Activity

Cho et al. isolated multiple bioactive compounds from *C. lanceolata* roots using the n-BuOH-soluble fraction of the ethanolic extract. Liquid chromatography-mass spectrometry (LC-MS) and high-resolution (HR)-LC-MS analyses revealed the presence of alkaloids and phenolic glycosides, such as tangshenosides and phenylpropanoids. Subsequent purification via preparative HPLC led to the identification of a new indole alkaloid N-glycoside (deodeokaloid) and three known phenolic compounds, tangshenoside I, tangshenoside IV, and chlorogenic acid. When these isolates were evaluated for their anti-*Helicobacter pylori* and antioxidant properties, tangshenoside IV exhibited notable antibacterial activity, with 36.8% inhibition of *H. pylori* growth. Chlorogenic acid demonstrated the strongest antioxidant capacity, effectively scavenging ABTS (1624.7 mmol TE/mol) and DPPH (707.5 mmol TE/mol) radicals. Moreover, tangshenosides I and IV and chlorogenic acid significantly reduced intracellular ROS levels in LPS-stimulated RAW 264.7 macrophages. Together, these findings highlight *C. lanceolata* roots as a promising natural source of antioxidant and anti-*H. pylori* bioactive constituents [15] (Table 5).

He et al. found that the fermentation of *C. lanceolata* with *Lactobacillus acidophilus* ADH, *Bifidobacterium longum* B6, *Lactobacillus rhamnosus* GG, or *Lactobacillus paracasei* at 37 °C for 10 days, followed by 500 MPa at 50 °C for 30 min could improve the extract yield and influence bioactivity. *B. longum* fermented by high-pressure extraction (BLF-HPE) showed the highest antimicrobial activity (minimum inhibitory concentration (MIC) < 14 mg/mL) against *Listeria monocytogenes*, *Staphylococcus aureus*, *Shigella boydii*, and *Salmonella typhimurium*. High-pressure processing of non-fermented samples increased the TPC (13.3 mg GAE/g) and enhanced antioxidant activity. In addition, BLF-HPE and *L. rhamnosus*-fermented high-pressure extracts exhibited superior antimutagenic activities against *S. typhimurium* TA100, with 82% and 83% inhibition, respectively [33] (Table 5). Another follow-up study by a similar group used high-pressure treatment (400 MPa, 20 min) followed by fermentation with *Bifidobacterium longum* B6 (HPE-BLF) and *L. rhamnosus* (HPE-LRF) at 37 °C for 7 days and found that the phenol amounts were significantly improved compared to conventional extraction (more than 8 mg GAE/g vs. 6.69 mg GAE/g), with lower flavonoid content. HPE-BLF and HPE-LRF showed the highest DPPH radical scavenging activity (EC_50_ = 1.26 and 1.18 mg/mL respectively), possessed excellent antimicrobial activity against *Staphylococcus aureus*, *Listeria monocytogenes*, *Salmonella typhimurium*, and *Shigella boydii* (MICs < 15 mg/mL), and inhibited α-glucosidase and tyrosinase activity. In vivo administration of HPE-BLF and HPE-LRF improved cognitive impairment in SCP-induced ICR mice. In addition, in vitro experiments using HEK293 cells demonstrated lower toxicity than that of conventional extracts. The results showed the importance of the extraction and fermentation processes for enhancing the bioactive potential of *C. lanceolata* [31] (Table 5). Jung et al. also observed the beneficial effects of fermentation with *L. rhamnosus* in improving total phenols and flavonoids in *C. lanceolata*, which improved its antioxidant, antibiofilm, and anticholinesterase activities [32] (Table 5). However, another study demonstrated the fermentation of *C. lanceolata* roots with *Lactobacillus acidophilus* against influenza A virus (IAV) using both in vitro and in vivo models. Fermentation increased the TPC (24.3 ± 0.15 mg/g) and crude saponin content (94.5 ± 5.18 mg/g) compared with the non-fermented extract (2.6 ± 1.27 mg/g and 50.3 ± 8.45 mg/g, respectively). However, lancemaside A content decreased from 103.3 ± 4.32 mg/kg to 5.7 ± 0.31 mg/kg. In addition, fermentation also enhanced antioxidative effects of *C. lanceolata* as shown by ferric reducing capacity, and DPPH (IC_50_ = 194.1 μg/mL) and ABTS scavenging activity (IC_50_ = 161.2 μg/mL). Treatment of IAV-infected A549 cells with fermented *C. lanceolata* extract (FCLE) reduced cytopathic effects and nucleocapsid proteins. It also reduced viral titers in MDCK cells in the post-treatment group. Fermented *C. lanceolata* effectively inhibited neuraminidase activity and hemagglutinin. Moreover, the FCLE administration in BALB/c mice increased survival rate to 100%, and reduced virus titer, attenuated lung histopathological changes, and reduced keratinocyte chemoattractant (KC) and IL-1β levels in BALF, thereby preventing lethal infection [19] (Table 5). These studies suggest that fermentation enhances the pharmacological potential of *C. lanceolata* by improving its phytochemical composition and biological activity.

**Table 5:** In vitro and in vivo studies of *C. lanceolata* and bioactive compounds in anti-microbial activity.

Compound/Extract	Model	Treatment	Findings	Ref.
Compounds isolated from the n-butanol root fraction (C1–C4)	Chemical based antioxidant assays	Compound C1–4 or PC (Trolox)	ABTS and DPPH radical scavenging activity (C4 > C1 > C2 > C3)	[15]
	Anti-*Helicobacter pylori* activity	*H. pylori* strain 51 ± compounds C1–C4 (100 μM) or metronidazole/quercetin	Anti-*H. pylori* activity (C3 > C2 > C1 > C4)	
	RAW264.7 cells	Compounds C1–4 (100 μM) ± LPS, 24 h	↓ Intracellular ROS (C2 > C3 > C4)	
*C. lanceolata* fermented root extract	Antioxidant assays	Non-fermented and probiotic-fermented *C. lanceolata* root extracts prepared by conventional or high-pressure extraction	↑ Antibacterial activity against *L. monocytogenes*, *S. aureus*, *Sh. boydii*, and *S. typhimurium* (highest in *B. longum*-fermented extract), ↑ total polyphenol content (highest in *L. paracasei*-fermented extract), ↑ DPPH radical scavenging and reducing power (highest in non-fermented high-pressure extract), and ↑ anti-mutagenic activity (highest in *B. longum*-fermented extract)	[33]
*C. lanceolata* fermented extract	Antioxidant assays	Non-fermented and probiotic-fermented *C. lanceolata* root extracts prepared by conventional or high-pressure extraction	↑ Total polyphenol content (highest in *L. rhamnosus*-fermented extract), ↑ phenolic acids (highest in *B. longum*-fermented extract), ↑ DPPH radical scavenging activity, ↑ Fe^2+^ chelating activity (highest in non-fermented high-pressure extract), ↑ antimicrobial activity against *S. aureus*, *L. monocytogenes*, *S. typhimurium*, and *S. boydii*, and ↑ α-glucosidase and tyrosinase inhibitory activities	[31]
	HEK293 cells	Fermented extract (100 μL), 48 h	↓ Cytotoxicity (lowest in *B. longum*-fermented extract)	
	ICR mice	SCP (1 mg/kg) ± fermented and non-fermented extract (conventional or high-pressure extraction), 667 mg/kg/day, 24 h, *n* = 10/group, OA	↑ Step-through latency, ↑ cognition, and ↑ memory (highest in *L. rhamnosus*-fermented extract)	
*C. lanceolata* fermented extract	Antioxidant assays	Non-fermented and *L. rhamnosus*-fermented *C. lanceolata* extracts fractionated into hexane, trichloromethane, ethyl acetate, butanol, and water fractions	↑ Total polyphenol content and FRAP activity (highest in fermented trichloromethane fraction), ↑ Fe^2+^ reducing activity (highest in non-fermented trichloromethane fraction), ↑ DPPH radical scavenging activity (trichloromethane and ethyl acetate fractions most active), ↑ antimicrobial activity against *L. monocytogenes*, *S. aureus, S. typhimurium*, and *S. boydii* (fermented extracts > non-fermented extracts), ↑ anti-biofilm activity against *S. aureus*, and ↑ AChE inhibitory activity (trichloromethane and ethyl acetate fractions most active)	[32]
*C. lanceolata* fermented root extract	Antioxidant assays	Fermented or non-fermented extract (10–1000 µg/mL)	↑ Total polyphenol content, ↑ saponins, ↑ reducing sugars, ↑ DPPH and ABTS radical scavenging activity, ↑ FRAP activity	[19]
	A549 cells	Fermented or non-fermented extract (3–3000 µg/mL) ± IAV	↓ IAV-induced cytopathic effects, ↓ viral nucleocapsid protein	
	MDCK cells	Fermented or non-fermented extract (30 and 100 µg/mL) ± IAV or oseltamivir	↓ Virus titer	
	cRBC	Fermented or non-fermented extract (30–1000 µg/mL) ± IAV	↓ cRBC aggregation (hemagglutination inhibition)	
	NA activity assay	Fermented or non-fermented extract (10–300 µg/mL) or oseltamivir	↓ NA activity	
	BALB/c mice	IAV ± fermented (10 and 100 mg kg) or non-fermented extract (100 mg/kg), every two days, total three treatments or oseltamivir, (*n* = 5/group)	↑ Survival rate, ↓ viral titer, ↓ lung pathological alterations, ↓ airway, vascular, and parenchymal inflammation score, ↓ KC, ↓ IL-1β, ↓ IFN-γ (ns)	

**Abbreviations:** ↑, increase; ↓, decrease; PC, positive control; DMSO, dimethyl sulfoxide; LPS, lipopolysaccharide; ABTS, 2,2′-azino-bis-(3-ethylbenzothiazoline-6-sulfonic acid); DPPH, 2,2-diphenyl-1-picrylhydrazyl; ROS, reactive oxygen species; SCP, scopolamine; OA, oral administration; FRAP, ferric reducing antioxidant power; AChE, acetylcholinesterase; NA, neuraminidase; IAV, influenza A virus; KC, keratinocyte chemoattractant; IL-1β, interleukin-1 beta; IFN-γ, interferon gamma; and ns, non-significant.

### 4.10. Immune Modulation

Macrophages play a central role in innate immunity by recognizing, engulfing, and eliminating pathogens while orchestrating inflammatory and tissue repair processes. Through the secretion of cytokines and growth factors, they bridge the innate and adaptive immune responses and maintain immune homeostasis [90].

An in vitro study by Lee et al. reported the regulatory roles of total MeOH extracts from fresh leaves (l-TME) and roots (r-TME) of *C. lanceolata*. The expression of pro-inflammatory mediators NO, TNF-α, IL-3, and IL-6 was found to be downregulated in l-TME and r-TME-exposed LPS-activated RAW264.7 cells. The rapid acceleration of fibrosarcoma kinase (Raf)-ERK signaling, which is responsible for the post-translational modification of pro-inflammatory gene products, was strongly blocked by these extracts. The classically activated inflammatory markers, CD80 and CD86, were also downregulated by these extracts. Furthermore, l-TME downregulated LPS-mediated phagocytic uptake, whereas r-TME upregulated it. Cell–cell clustering by CD43 and CD29 was blocked by l-TME, while cell–cell aggregation by CD43 and CD29 was induced by r-TME in U937 cells [27] (Table 6).

Hematopoietic cytokines are a group of signaling molecules that play essential roles in regulating the proliferation, differentiation, and survival of blood-forming cells within the bone marrow. They act as key mediators in the maintenance of hematopoietic homeostasis and orchestrate immune responses by influencing the production of various leukocyte lineages. Byeon et al. reported the immunomodulatory properties of *C. lanceolata* in vitro by enhancing the expression of hematopoietic cytokines, such as granulocyte-macrophage colony-stimulating factor (GM-CSF) in RAW264.7 cells. The BuOH fraction of *C. lanceolata* significantly enhanced splenocyte proliferation in murine splenocytes and increased both NF-κB and activator protein (AP)-1-induced luciferase activities in HEK293 cells. Furthermore, ERK and PI3K were involved in *C. lanceolata*-mediated activation of transcription factors [91] (Table 6).

Another in vitro study by Byeon and co-workers showed immunomodulatory roles of saponin fractions of *C. lanceolata* in LPS-induced RAW264.7 cells [28]. The saponin fraction downregulated NO and TNF-α production, together with reducing dextran uptake (phagocytosis). Saponin fraction also suppressed iNOS mRNA expression, and IκBα phosphorylation in LPS-induced RAW264.7 cells. The saponin fraction blocked U937 cell–cell adhesion, and suppressed concanavalin A-induced splenic lymphocyte proliferation and Th1 cytokine IFN-γ. Also, the saponin fraction suppressed degradation in RBL-2H3 cells by suppressing the β-hexosaminidase release [28] (Table 6).

Joh and Kim demonstrated the anti-inflammatory potential of lancemaside A in both in vitro and in vivo models using LPS-induced peritoneal macrophages and ICR mice. Lancemaside A suppressed the expression of key inflammatory mediators, including IL-1β, TNF-α, iNOS, COX-2, PGE2, and NO, in LPS-stimulated peritoneal macrophages. In addition, it reduced IKK-β phosphorylation, prevented IκB-α degradation, decreased p-p65/p65 levels, and inhibited the nuclear translocation of NF-κB p65, collectively attenuating NF-κB-mediated inflammatory signaling. In addition, treatment with lancemaside A reduced p-ERK expression. Lancemaside A also inhibited the interaction between TLR4 and LPS, thereby inhibiting IRAK-4 activation. The in vivo data supported the in vitro findings as lancemaside A administration in LPS-induced ICR mice downregulated the expression of IL-1β and TNF-α [10] (Table 6). In an in vitro study, lancemaside A was also found to reduce LPS-induced pro-inflammatory mediators such as NO, TNF-α, and IL-6, along with phosphorylated JNK, and ROS by suppressing the expression of NADPH oxidase subunits such as neutrophil cytosolic factor 1 (NCF1 or p47^phox^), neutrophil cytosolic factor 2 (NCF2 or p67^phox^), and glycoprotein 91^phox^ or NADPH oxidase 2 (NOX2 or gp91^phox^), and phosphorylation of p47^phox^ in BV-2 microglial cells. In addition, it increased HO-1 expression and neuronal cell viability in BV-2 and Neuro-2a co-culture systems [92] (Table 6). Joh et al. demonstrated the inhibitory effects of echinocystic acid in a 12-O-tetradecanoylphorbol-13-acetate (TPA)-induced in vivo dermatitis model. The topical administration of echinocystic acid was found to suppress ear swelling, myeloperoxidase activity, and expression of pro-inflammatory mediators COX-2, iNOS, TNF-α, IL-1β, and NF-κB in TPA-induced mouse ears and NF-κB in LPS-exposed peritoneal macrophages [93] (Table 6). Lancemaside A was also shown to reduce NO production and mRNA expression levels of iNOS and CD80, along with reducing morphological alterations, phagocytosis, and ROS generation in LPS-induced RAW264.7 cells. In addition, it also reduced cell–cell aggregation and cell–fibronectin adhesion and reduced TNF-α induced intercellular adhesion molecule (ICAM)-1, p-p65, p-IκBα, and kinase activity of IKKα/β in U937 cells [9] (Table 6).

Boo et al. reported the immune cell, nitrite, and ABTS radical scavenging activities of ethanolic extracts (30%, 50%, 70%, and 100% EtOH) of *C. lanceolata* from six Korean districts (Hwasun, Uljin, Hoengseong, Jeju, Jecheon, and Muju). The 50% EtOH extract of *C. lanceolata* exhibited promising immune activity by enhancing human T cell growth, whereas the 30% EtOH extract of *C. lanceolata* exhibited the highest human B cell growth. The 30% EtOH extract also showed the highest NO^−^_2_ scavenging activity at a pH of 1.2. The ABTS radical scavenging activity assay showed that the 100% EtOH extract was the most potent among other concentrations, with 100% ABTS radical scavenging activity. Although the immune activity and NO^−^_2_ scavenging activity showed no correlation with the selected regions, the extracts from the Jeju and Jecheon regions showed slightly higher activity [94] (Table 6).

The addition of *C. lanceolata* powder in traditional rice cakes was also found to enhance physical and chemical properties (pH, moisture, crude protein, crude fat and total flavonoids) and increase DPPH radical scavenging activity (IC_50_ = 119.01 mg/mL vs. IC_50_ = 844.26 mg/mL) as compared with traditional rice cakes, and showed anti-fungal properties [95] (Table 6). Lee et al. found an abundance of lancemaside A in the n-butanol fraction of *C. lanceolata* using LC/tandem mass spectrometry (MS/MS) and carbon-13 nuclear magnetic resonance spectroscopy (^13^C-NMR) values and observed the regulatory role of lancemaside A in nitric oxide synthesis in vitro using human umbilical vein endothelial cells (HUVECs). In addition, lancemaside A was observed to enhance p-eNOS and p-Akt and even prevent N-nitro-L-arginine methyl ester (L-NAME, an eNOS inhibitor)-associated reduction. The inhibitors LY294002 (PI3K inhibitor) and A6730 (Akt1/2 kinase inhibitor) reduced p-eNOS expression; however, co-treatment with lancemaside A reversed these inhibitor-mediated effects, revealing the role of lancemaside A in activating the PI3K/Akt/eNOS signaling pathway [96] (Table 6).

A 2021 study demonstrated the effects of extraction solvents on the pharmacological potential of *C. lanceolata* [30]. The 30% EtOH, 90 °C hot water, 70 °C hot water, and DW extract was prepared and analyzed for TPC and TFC. The 30% EtOH extract had the highest TPC followed by 90 °C hot water, 70 °C hot water, and DW extract. The TFC showed a trend similar to that of TPC, with the 30% EtOH extract showing the highest TFC. The extract obtained using 30% EtOH exhibited the highest DPPH and ABTS scavenging activities. In vitro studies using RAW264.7 cells revealed that the extract was non-toxic at concentrations up to 200 μg/mL and inhibited LPS-induced NO production. Also, the 30% EtOH extract was found to be the most potent anti-inflammatory agent as it downregulated LPS-induced pro-inflammatory cytokines such as TNF-α, IL-6, and IL-1β. These results suggested that *C. lanceolata* possesses promising antioxidant and anti-inflammatory potential [30] (Table 6).

Fermented aerial tissues (F-CSA) and roots (F-CSR) of *C. lanceolata* produced using *Aspergillus oryzae*, together with non-fermented SMART farm aerial tissues (CSA), roots (CSR), whole plants (CSW), and outdoor-grown roots (CLR), were extracted using 70% EtOH. The resulting extracts were analyzed using UPLC coupled with electrospray ionization quadrupole time-of-flight mass spectrometry (UPLC-ESI-QTOF-MS) for metabolite profiling, followed by quantitative determination of luteolin and kaempferol using HPLC and measurement of total polyphenols, tannins, flavonoids, and saponins. F-CSA exhibited the highest TPC (57.00 mg GAE/g) and tannin content (36.27 mg GAE/g). In addition, F-CSA showed the highest TFC (26.17 mg CE/g) compared with non-fermented CSA. Total saponins were detected in all the samples, with CSR and CLR exhibiting the highest levels. Quantitative analysis revealed the presence of luteolin and kaempferol exclusively in the aerial parts of F-CSA and CSA. Notably, F-CSA contained substantially higher levels of luteolin (7.83 ± 0.16 mg/g) than CSA (1.71 ± 0.36 mg/g). These findings indicate that fermentation substantially alters the phytochemical profile of *C. lanceolata* aerial tissues. In vitro analyses demonstrated that all samples were non-toxic to RAW264.7 cells at concentrations up to 1000 µg/mL. Furthermore, F-CSA inhibited LPS-induced NO production in RAW264.7 cells. Pre-treatment with F-CSA reduced NO production, iNOS, PGE2, COX-2, and the expression of pro-inflammatory cytokines IL-1β, TNF-α, and IL-6 in LPS-stimulated RAW264.7 cells. In addition, F-CSA decreased LPS-induced phosphorylation of p65 and JNK. These in vitro findings were further supported by in vivo experiments, in which F-CSA increased the survival rate and reduced NO and IL-6 levels in LPS-induced CD1 mice [29] (Table 6). A metabolomic study using UPLC-QTOF-MS examined *C. lanceolata* plants cultivated in three different regions of Korea (Hoengseong, Jeonseon, and Jeju). This study demonstrates regional variations in metabolite composition and antioxidant activity. Plants grown in Jeju showed higher levels of primary metabolites, and plants grown in Hoengseong and Jeonseon showed higher levels of secondary metabolites (phenylpropanoids and triterpenoid saponins) with higher bioactive compound content and antioxidant capacity, suggesting that the cultivation region may influence the phytochemical composition and bioactive potential of *C. lanceolata* [16] (Table 6).

Age-related macular degeneration (AMD) is a leading cause of irreversible vision loss in older adults and is characterized by the progressive degeneration of the retinal pigment epithelium (RPE), photoreceptors, and Bruch’s membrane. One of the key pathological mechanisms implicated in AMD development is carbonyl stress, which refers to the accumulation of reactive carbonyl compounds (RCCs), such as methylglyoxal, glyoxal, and 4-hydroxynonenal. Carbonyl stress leads to the formation of advanced glycation end products (AGEs) and advanced lipid oxidation end products (ALEs), which modify cellular proteins, lipids, and DNA in the retina. These modifications induce OS, inflammation, and mitochondrial dysfunction in the RPE cells, contributing to drusen formation and retinal degeneration. Elevated levels of carbonyl stress markers have been detected in patients with AMD, highlighting their roles as critical mediators of retinal aging and disease progression. Recently, an in vitro study was conducted to investigate the protective effects of *C. lanceolata* in an ARPE-19 cell model of AMD. The treatment activated the Keap1/Nrf2/HO-1 pathway, suppressed 4-hydroxynonenal, increased Bcl-2 and Bcl-XL expression, and decreased the pro-apoptotic protein Bcl-2 interacting mediator of cell death (Bim) [97] (Table 6).

**Table 6:** In vitro and in vivo studies of *C. lanceolata* and bioactive compounds in immune modulation.

Compound/ Extract	Model	Treatment	Findings	Ref.
*C. lanceolata* leaves or root methanolic extracts	RAW264.7 cells	LPS (2.5 µg/mL) ± root or leaves extract (50–400 µg/mL), 30 min, or 6 or 24 h	↓ NO, ↓ TNF-α, ↓ IL-3, ↓ IL-6, ↓ Raf/ERK signaling, ↓ CD80, ↓ CD86, ↓ dectin-1, ↓ β-glucan-induced NO production, and↓ phagocytosis (leaf extract); ↑ phagocytosis (root extract)	[27]
	U937 cells	Root or leaf extract (1 h) followed by activation with agonistic antibodies, 1 µg/mL, 2h	↓ CD43/CD29-mediated cell clustering and ↑ fibronectin adhesion (leaf extract); ↑ CD43/CD29-mediated cell clustering (root extract)	
*C. lanceolata* root butanolic fraction	RAW264.7 cells	LPS (2 µg/mL) ± root fraction (0–200 µg/mL) or comparative fractions of *C. lanceolata*, 100 µg/mL, 30–60 min	↑ GM-CSF, ↑ p-IκBα, ↑ p-ERK, ↑ p-p38, ↑ p-p85, ↑ TNF-α	[91]
	C57BL/6 mouse splenocytes	LPS or Con A ± root fraction (0–200 µg/mL), 48 h	↑ Splenocyte proliferation	
	HEK-293 cells	NF-κB/AP-1 luciferase reporter assay; rest similar as splenocytes	↑ NF-κB- and AP-1-mediated luciferase activities	
*C. lanceolata* saponin fraction	RAW264.7 cells	Different butanolic derived fractions (0–50 µg/mL) or saponin fraction (12.5–50 µg/mL) ± LPS, SNP, or FITC-dextran, 6 h or 24 h, or 5 or 30 min	↓ NO (SF only), ↓ TNF-α, ↓ phagocytosis, ↓ iNOS, ↓ p-IκBα	[28]
	U937 cells	Saponin fraction (12.5–100 µg/mL) + CD29/CD43 agonistic antibodies (1 µg/mL), 2h	↓ CD29/CD43-mediated cell adhesion	
	Splenocytes	Saponin fraction (25–50 µg/mL) ± Con A, LPS, or PHA	↓ T-cell proliferation (Con A > PHA) and ↓ IFN-γ	
	RBL-2H3 cells	Saponin fraction (50–300 µg/mL) + DNP-BSA (IgE-sensitized)	↓ β-hexosaminidase release	
Lancemaside A	Peritoneal macrophages	LPS (1 µg/mL) ± Lancemaside A (2–10 µM, 20 h.	↓ IL-1β, ↓ TNF-α, ↓ iNOS, ↓ COX-2, ↓ PGE2, ↓ NO	[10]
		LPS (50 ng/mL) ± Lancemaside A (10 µM), 0–60 min	↓ p-IKK-β, ↓ IκB-α degradation, ↓ p-p65, ↓ NF-κB p65 nuclear translocation, ↓ pERK, ↓ p-JNK, ↓ p-p38, ↓ LPS and TLR4 interaction, ↓ LPS-macrophage binding, ↓ IRAK4	
	ICR mice	LPS (4 mg/kg) ± Lancemaside A (5 mg/kg), 4 h (*n* = 6), not given	↓ IL-1β, ↓ TNF-α	
Lancemaside A	BV 2 cells	Lancemaside A (0–20 µM) or SP600125 (10–20 µM) ± LPS (100 ng/mL)	↓ NO, ↓ TNF-α, ↓ IL-6, ↓ iNOS, ↓ COX-2, ↓ pJNK, ↓ NF-κB & ↓ AP-1 DNA-binding activity, ↓ ROS, ↓ p47^phox^, ↓ p67^phox^, ↓ gp91^phox^, ↓ p-p47^phox^, ↑ HO-1	[92]
	BV-2 coculture with Neuro-2a cells	Lancemaside A (10–20 µM) or SP600125 (10–20 µM) ± LPS-stimulated BV-2/Neuro-2a coculture	↑ Cell viability	
Echinocystic acid	ICR mice	TPA (3 μg) ± echinocystic acid (0.05 or 0.10%) or dexamethasone, topical administration (*n* = 6/group)	↓ Ear-swelling and thickness, ↓ neutrophil accumulation, ↓ edema formation, ↓ MPO activity, ↓ COX-2, ↓ iNOS, ↓ TNF-α, ↓ IL-1β, ↓ p-p65	[93]
	Peritoneal macrophages	Echinocystic acid (2–10 μM) ± LPS (50 ng/mL) 90 min	↓ p-p65, ↓ NF-κB activation and translocation	
Lancemaside A	RAW264.7 cells	Lancemaside A (0–30 µM) ± LPS (1 µg/mL) or SNP (10 mM)	↓ NO, ↓ iNOS, ↓ CD80, ↓ morphological alteration	[9]
	U937 cells	Lancemaside A (15–30 µM) or BAY11-7082 + FITC-dextran or SNP Scratch-wound ± Lancemaside A Lancemaside A (0–30 µM) or BAY11-7082 + anti-CD29 antibody, fibronectin, or TNF-α	↓ Phagocytosis, ↓ ROS generation, ↓ Cell aggregation, ↑ cell detachment, ↓ fibronectin adhesion, ↓ ICAM-1, ↓ p-p65, ↓ p-IκBα, ↓ IKKα/β kinase activity	
*C. lanceolata* ethanolic extracts from different Korean regions	Antioxidant assays	Extracts (30%, 50%, 70%, and 100%; 0.5–20 mg/mL) under different pH conditions	↑ Nitrite scavenging (highest in 30% at pH 1.2) and↑ ABTS radical scavenging (highest in 100%)	[94]
	T cell and B cell	Extracts (30%, 50%, 70%, and 100%) from different Korean regions (0.5 µg/mL)	↑ T-cell activity (50% highest), ↑ B-cell activity (30% highest), and ↑ cell growth	
*C. lanceolata* powder fused rice cake	Antioxidant, Antifungal and Antistaling activity assays	Control rice cake or *C. lanceolata*-fortified rice cake (0–100 mg/mL)	↑ Crude protein, ↑ crude fat, ↑ total flavonoid content, ↑ moisture, ↑ DPPH scavenging, ↑ anti-fungal activity, ↑ antistaling activity	[95]
Lancemaside A	HUVECs	Lancemaside A (0.5–15 µM) ± L-NAME (25 µM), LY294002 (25 µM), or A6730 (5 µM)	↑ NO release, ↑ eNOS, ↑ p-eNOS, ↑ p-Akt	[96]
*C. lanceolata* extracts	Antioxidant assays	30% ethanolic extract or water extracts (distilled water, 70 °C, and 90 °C), 0.5–10 mg/mL	Total polyphenol and flavonoid content (30% > 90 °C > 70° > water), ↑ DPPH and ABTS radical scavenging activity (30% > 90 °C)	[30]
	RAW264.7 cells	Extract (50–400 µg/mL) ± LPS (0.5 µg/mL)	↓ NO,↓ TNF-α, ↓ IL-6, ↓ IL-1β (30% extract, most prominent)	
*C. lanceolata* fermented sprouts	Chemical assays	*C. lanceolata* fermented with A. oryzae	Highest total polyphenol, tannin and flavonoid content fermented aerial-part extract; ↑ luteolin and kaempferol in fermented and non-fermented aerial-part extracts	[29]
	RAW264.7 cells	Fermented and non-fermented aerial-part, root, and whole-plant extracts (125–1000 µg/mL) ± LPS (1 µg/mL)	↑ Cell viability, ↓ NO, ↓ iNOS, ↓ PGE2, ↓ COX-2, ↓ IL-6, ↓ IL-1β, ↓ TNF-α, ↓ p-p65, ↓ p-JNK	
	CD1 mice (ICR mice)	Fermented aerial-part extract (50–200 mg/kg) ± LPS (25 mg/kg i.p.) (*n* = 12/group), OA	↑ Survival rate, ↓ NO, ↓ IL-6	
*Codonopsis lanceolata* ethanolic extracts from different regions	Antioxidant assays		↑ DPPH radical scavenging activity,↑ FRAP activity,↑ antioxidant activity	[16]
	HepG2 cells	Extracts (500 μg/mL) ± H_2_O_2_ (500 μM)	↓ ROS	
*C. lanceolata*	ARPE-19 cells	A2E (30–40 μM) and blue light exposure ± *C. lanceolata* (5–100 μM) or lutein (25 μM)	↓ Cell death, ↓ apoptosis, ↑ Keap1, ↑ Nrf2, ↑ HO-1, ↓ 4-HNE, ↑ Bcl-2, ↑ Bcl-XL, ↓ Bim	[97]

Abbreviations: ↑, increase; ↓, decrease; LPS, lipopolysaccharides; NO, nitric oxide; TNF-α, tumor necrosis factor alpha; IL, interleukin; p, phosphorylated; Raf, rapidly accelerated fibrosarcoma; ERK, extracellular signal-regulated kinase; CD, cluster of differentiation; Con A, concanavalin A; NF-κB, nuclear factor kappa-light-chain-enhancer of activated B cells; AP-1, activator protein-1; GM-CSF, granulocyte-macrophage-colony stimulating factor; IκB-α, inhibitor of kappa B-alpha; SNP, sodium nitroprusside; FITC, fluorescein isothiocyanate; PHA, phytohemagglutinin; DNP-BSA, 2,4-dinitrophenyl-bovine serum albumin; iNOS, inducible nitric oxide synthase; IFN-γ, interferon gamma; COX-2, cyclooxygenase-2; PGE2, prostaglandin E2; IKK-β, inhibitor of nuclear factor kappa-B kinase subunit beta; JNK, c-Jun N-terminal kinase; TLR4, toll like receptor 4; IRAK4, interleukin-1 receptor-associated kinase 4; ROS, reactive oxygen species; HO-1, heme oxygenase-1; TPA, 12-*O*-tetra decanoylphorbol-13-acetate; MPO, myeloperoxidase; ICAM-1, intercellular adhesion molecule-1; ABTS, 2,2′-azino-bis(3-ethylbenzothiazoline-6-sulfonic acid); DPPH, 2,2-diphenyl-1-picrylhydrazyl; L-NAME, Nω-nitro-L-arginine methyl ester; eNOS, endothelial nitric oxide synthase; Akt, protein kinase B; OA, oral administration; H_2_O_2_, hydrogen peroxide; FRAP, ferric reducing antioxidant power; A2E, bis-retinoid N-retinyl-N-retinylidene ethanolamine; Keap1, Kelch-like ECH-associated protein 1; Nrf2, nuclear factor erythroid-2-related factor 2; 4-HNE, 4-hydroxynonenal; Bcl-2, B cell lymphoma 2; Bcl-XL, B-cell lymphoma extra-large; and Bim, Bcl-2 interacting mediator of cell death.

## 5. Common Molecular Mechanisms Underlying the Pharmacological Effects of *Codonopsis lanceolata*

**Figure 4:**
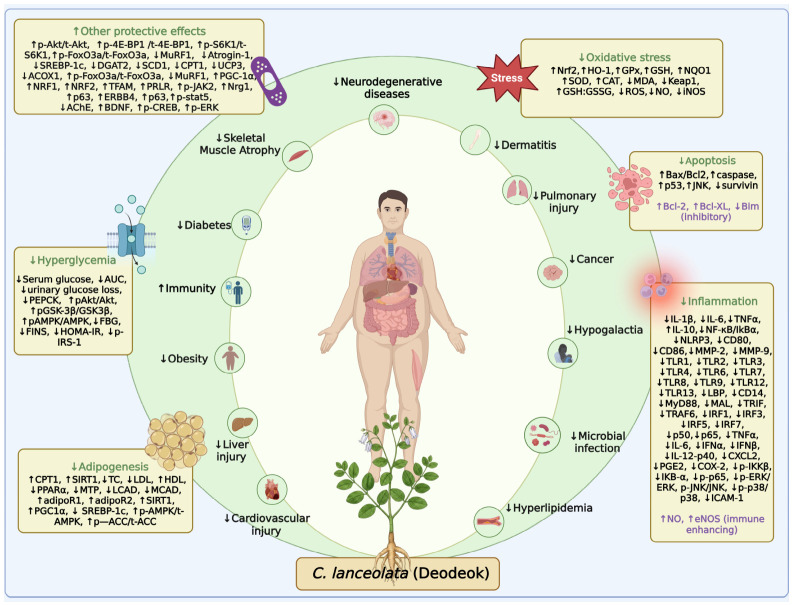
Systemic pharmacological effects of *Codonopsis lanceolata* (Deodeok) on human health. This figure summarizes the diverse therapeutic activities reported for *C. lanceolata* and its bioactive compounds. Documented effects include attenuation of oxidative stress, inflammation, apoptosis, cancer progression, pulmonary injury, hyperlipidemia, microbial infection, hypogalactia, cardiovascular injury, liver injury, obesity, diabetes, dermatitis, skeletal muscle atrophy, and neurodegenerative processes. The figure also highlights improvements in immunity and glycemic regulation, along with suppression of adipogenesis. Key molecular targets and pathways, such as Akt, AMPK, NF-κB, MAPK, JNK, ERK, apoptotic regulators, antioxidant enzymes, and metabolic mediators, are listed to summarize molecular pathways reported in experimental studies. Collectively, the schematic depicts the broad, multi-organ protective potential of *C. lanceolata*, supporting its relevance as a functional food and medicinal plant. ↑, increase; ↓, decrease. Created in BioRender. Lee, H. (2026) https://BioRender.com/ifjxatu (accessed on 5 June 2026).

Although the pharmacological activities of *C. lanceolata* have been investigated in diverse disease models, many of the reported therapeutic effects converge on several common molecular mechanisms. After discussing disease-specific evidence, this section summarizes the major signaling pathways and biological processes that collectively underlie the pharmacological activities of *C. lanceolata*. Figure 4 illustrates the principal molecular networks and signaling pathways commonly modulated by *C. lanceolata* and its representative bioactive constituents.

### 5.1. Regulation of Oxidative Stress and Antioxidant Defense

The regulation of OS is one of the most consistently reported mechanisms underlying the pharmacological activities of *C. lanceolata*. Across diverse disease models, including metabolic, neurodegenerative, pulmonary, cardiovascular, and ocular disorders, *C. lanceolata* has been shown to reduce oxidative damage while enhancing endogenous antioxidant defense systems [7,21,25,65,66,79,81,97]. Several studies have shown that *C. lanceolata* extracts, polysaccharides, and fermented preparations reduce intracellular ROS generation and lipid peroxidation while enhancing endogenous antioxidant defense [21,30,31,32,33,38,97]. In metabolic disease models, *C. lanceolata* polysaccharides improved antioxidant enzyme activities, including SOD and CAT, reduced MDA levels, and restored the glutathione/glutathione disulfide (GSH/GSSG) balance [21]. These findings suggest that the antioxidant effects of *C. lanceolata* may involve Nrf2-associated signaling pathways, which are known to regulate antioxidant mediators such as HO-1 and NQO1 and contribute to cellular redox homeostasis [21,31,33,97]. Consequently, these changes may reduce lipid peroxidation, mitochondrial dysfunction, protein oxidation, and other oxidative injuries that contribute to chronic disease progression. Similar antioxidant-related protective effects have been reported in neuroprotective, hepatic, pulmonary, and ocular models, in which *C. lanceolata* preparations reduced oxidative injury and improved cell survival [6,7,16,25,79,81,86,88,97]. Additional studies have demonstrated the strong radical-scavenging and antioxidant capacities of *C. lanceolata* extracts prepared under different geographical origins and processing conditions, supporting the broad antioxidant potential of this plant [16,30,38,95]. Collectively, these findings suggest that the reinforcement of antioxidant defense, particularly through Nrf2-associated signaling, may represent an important mechanism underlying the reported protective effects of *C. lanceolata*.

### 5.2. Suppression of Inflammatory Signaling Pathways

The modulation of inflammatory signaling pathways is another major mechanism contributing to the therapeutic effects of *C. lanceolata*. Anti-inflammatory activity has been consistently observed in macrophage-, pulmonary-, hepatic-, intestinal-, and immune-related disease models, in which *C. lanceolata* reduced the production of pro-inflammatory mediators and attenuated inflammatory tissue damage. The anti-inflammatory activity of *C. lanceolata* has been largely associated with inhibition of TLR4/NF-κB and MAPK signaling pathways. Chronic activation of these pathways promotes the production of proinflammatory cytokines and mediators that contribute to tissue injury and disease progression [83,84,85,86]. Lancemaside A inhibits LPS-induced inflammatory responses by targeting the LPS/TLR4 complex and suppressing downstream inflammatory signaling [10]. In macrophage and immune cell models, *C. lanceolata* extracts or saponin fractions decreased inflammatory mediators such as NO, TNF-α, IL-1β, IL-6, iNOS, COX-2, and PGE2 [10,27,28,29]. These effects were accompanied by reduced activation of NF-κB and MAPK-related proteins, including p38, ERK, and JNK [9,10,29,65,86,93]. Comparable anti-inflammatory effects have also been reported for different solvent extracts of *C. lanceolata*, which reduced NO, TNF-α, IL-1β, and IL-6 production in LPS-stimulated macrophages [30]. Similar mechanisms were observed in pulmonary inflammation models, where echinocystic acid or *C. lanceolata*-containing preparations suppressed LPS- or particulate matter-induced inflammatory responses through inhibition of TLR4/NF-κB/MAPK signaling [7,85,86,88]. Thus, the modulation of inflammatory signaling appears to be a common mechanism underlying the reported anti-inflammatory, pulmonary protective, hepatoprotective, and immunomodulatory effects of *C. lanceolata* [6,7,27,28,68,86,88].

### 5.3. Regulation of Metabolic Signaling and Energy Homeostasis

The regulation of metabolic homeostasis is another major mechanism underlying the pharmacological activities of *C. lanceolata* in diabetes, obesity, fatty liver disease, and sarcopenic obesity models. Accumulating evidence suggests that *C. lanceolata* improves glucose utilization, lipid metabolism, insulin sensitivity, and mitochondrial function through the coordinated modulation of multiple metabolic signaling pathways [21,39,52,68]. In diabetic and insulin-resistant models, *C. lanceolata* water extract and polysaccharide improved glucose metabolism and insulin sensitivity by modulating insulin signaling pathways, including Akt and GSK-3β phosphorylation [21,39]. In skeletal muscle atrophy and sarcopenic obesity models, *C. lanceolata* extract and tangshenoside I activated PI3K/Akt/mTOR signaling, suppressed muscle degradation markers such as MuRF1 and Atrogin-1, and improved mitochondrial biogenesis through SIRT1/PGC-1α-related signaling [26,52]. In liver injury and metabolic disease models, *C. lanceolata* improves lipid metabolism and attenuates hepatic metabolic abnormalities by regulating lipid metabolic pathways and associated signaling mediators [6,68]. These findings suggest that *C. lanceolata* exerts metabolic benefits by improving insulin sensitivity, enhancing fatty acid oxidation, suppressing lipogenesis, and maintaining mitochondrial function. Such coordinated regulation of glucose utilization, lipid metabolism, and cellular energy homeostasis may account for the beneficial effects of *C. lanceolata* in diabetes, obesity, fatty liver disease, and sarcopenic obesity models. In cardiovascular and endothelial models, *C. lanceolata* extract and lancemaside A improved vascular function by reducing blood pressure, suppressing NOX2/NF-κB/MAPK-related signaling, enhancing NO-related responses, and promoting vasorelaxation, including through eNOS/PI3K/Akt-associated pathways [23,65,66,67,96].

### 5.4. Modulation of Apoptosis and Cell Survival

The regulation of apoptosis and cell survival represents another important mechanism underlying the diverse biological activities of *C. lanceolata*. Interestingly, the effects of *C. lanceolata* on apoptotic pathways appear to be highly context-dependent, promoting apoptosis in malignant cells while protecting normal tissues from excessive cell death under pathological conditions. In cancer models, *C. lanceolata* extracts and isolated triterpenoid compounds promoted apoptosis by increasing pro-apoptotic mediators, such as Bax, Bak, JNK, and caspases, while reducing anti-apoptotic proteins, including Bcl-2 and survivin [8,20,56,57,58]. In addition, lancemaside A inhibited ovarian cancer cell migration and invasion through ROS-mediated p38 signaling and suppression of matrix metalloproteinases, including MMP-2 and MMP-9 [60]. These findings suggest that *C. lanceolata* exerts anti-cancer effects by promoting apoptotic signaling and limiting tumor progression. Additional studies have demonstrated the cytotoxic activity of various ethanol extracts against HeLa, Calu-6, and MCF-7 cancer cells, supporting the role of *C. lanceolata* in suppressing tumor cell survival [20,58]. In contrast, in non-cancer injury models, such as neurotoxicity, ischemic brain injury, and OS-related retinal damage, *C. lanceolata* preparations reduced excessive apoptosis and improved cellular survival through antioxidant and cytoprotective mechanisms [79,81,97]. These neuroprotective effects are associated with the preservation of antioxidant defenses and neurotrophic factors, including SOD1 and BDNF, thereby supporting neuronal survival under pathological conditions [81]. Therefore, *C. lanceolata* may either promote apoptosis in malignant cells or suppress pathological apoptosis in damaged normal tissues, depending on the biological context. This dual regulatory capacity highlights the context-dependent nature of *C. lanceolata*-mediated cell survival signaling.

### 5.5. Neurotrophic and Cholinergic Mechanisms

The regulation of neurotrophic signaling and cholinergic neurotransmission represents another important mechanism underlying the neuroprotective effects of *C. lanceolata*. Cholinergic dysfunction, OS, neuroinflammation, and reduced neurotrophic support are the major contributors to cognitive decline and neurodegenerative disorders. Several studies have demonstrated that *C. lanceolata* and its bioactive constituents improve cognitive function by modulating pathological processes. In experimental models of memory impairment, lancemaside A and its metabolite echinocystic acid ameliorated SCP-induced cognitive deficits and improved learning and memory performance [74]. Similarly, steamed and fermented *C. lanceolata* preparations significantly improved cognitive performance and attenuated memory impairment in both SCP- and amyloid-β-induced models [24,76,80]. These beneficial effects have been partly attributed to the modulation of cholinergic signaling pathways, including the inhibition of AChE activity, thereby enhancing cholinergic neurotransmission [32,74]. Several studies have demonstrated that the neuroprotective effects of *C. lanceolata* involve the activation of neurotrophic signaling pathways. Treatment with *C. lanceolata* extract increased the expression of BDNF and its downstream mediators, including CREB and ERK, which are essential for neuronal survival, synaptic plasticity, and memory formation [76,79,80,81]. In addition, lancemaside A has been reported to suppress microglial activation by modulating JNK signaling, suggesting a role in regulating neuroinflammatory responses associated with neurodegenerative disorders [92]. Furthermore, *C. lanceolata* reduced OS-induced neuronal damage and preserved antioxidant defense systems, including SOD1 activity, thereby contributing to improved neuronal viability and functional recovery [79,81]. Collectively, the available evidence suggests that *C. lanceolata* may exert neuroprotective effects through the coordinated modulation of cholinergic neurotransmission, neurotrophic signaling, neuroinflammatory responses, and antioxidant defenses. The modulation of AChE, BDNF/CREB/ERK-associated pathways, JNK signaling, and OS responses appear to represent a major mechanistic basis for the beneficial effects of *C. lanceolata* on cognitive dysfunction and neurodegenerative disorders.

### 5.6. Immunomodulatory, Endocrine, and Antimicrobial Mechanisms

The regulation of immune and endocrine signaling is another important mechanism that contributes to the diverse pharmacological effects of *C. lanceolata*. Increasing evidence indicates that *C. lanceolata* modulates both the innate and adaptive immune responses, thereby contributing to immune homeostasis and host defense [27,28,29,91]. Several studies have demonstrated that *C. lanceolata* extracts and saponin-rich fractions regulate macrophage-mediated immune responses by enhancing phagocytic activity and modulating the production of inflammatory cytokines and immune mediators [27,28,29,91]. In addition, *C. lanceolata* preparations have been shown to suppress excessive immune activation by reducing the expression of inflammatory mediators such as TNF-α, IL-6, NO, and other pro-inflammatory factors in activated immune cells [9,28,29]. These findings suggest that *C. lanceolata* contributes to the maintenance of immune homeostasis through balanced regulation of immune cell activation and cytokine signaling [94]. Immunomodulatory effects of *C. lanceolata* have also been observed in respiratory inflammatory disorders. In allergic lung inflammation models, *C. lanceolata* attenuated Th2-associated immune responses and reduced airway inflammation, partly through the regulation of cytokine production and enhancement of antioxidant defense mechanisms [7]. Similarly, fermented sprouts and other preparations suppressed inflammatory responses through modulation of NF-κB-associated signaling pathways [29]. In addition to immune regulation, *C. lanceolata* also demonstrates endocrine regulatory activity. A recent study showed that *C. lanceolata* polysaccharides ameliorated high-fat diet-induced postpartum hypogalactia through activation of the prolactin receptor (PRLR)/JAK2/STAT5 signaling pathway, thereby promoting mammary gland function and lactation-related responses [22]. These findings suggest that the biological activities of *C. lanceolata* may extend beyond its conventional anti-inflammatory effects and involve the regulation of hormone-responsive signaling pathways. Collectively, the available evidence suggests that *C. lanceolata* might exert immunomodulatory and endocrine regulatory effects through the coordinated modulation of macrophage function, cytokine signaling, inflammatory mediators, and PRLR/JAK2/STAT5-associated pathways. In addition, lancemaside A has been reported to enhance endothelial NO production through the activation of Akt/eNOS signaling, indicating that certain bioactive constituents of *C. lanceolata* may regulate vascular homeostasis through NO-dependent mechanisms [96]. These mechanisms may contribute to the reported biological effects on inflammatory, metabolic, respiratory, and reproductive disorders.

In addition to its immunomodulatory activity, several studies have reported direct antimicrobial effects of *C. lanceolata*. Fermented and fractionated preparations inhibited the growth of *Helicobacter pylori*, *Listeria monocytogenes*, *Staphylococcus aureus*, *Salmonella typhimurium*, and *Shigella boydii*, and exhibited antibiofilm activity. Furthermore, fermented *C. lanceolata* extracts attenuated influenza A virus infection by reducing viral titers, suppressing nucleocapsid protein expression, inhibiting neuraminidase activity, and improving the survival of infected mice. These findings suggest that beyond host immune regulation, *C. lanceolata* may exert direct antimicrobial actions that contribute to its overall therapeutic potential [15,19,31,32,33].

## 6. Safety, Toxicological Considerations, and Current Limitations

Although *C. lanceolata* has been consumed for centuries as a traditional food and medicinal plant, with a generally favorable safety profile, comprehensive toxicological investigations remain scarce. At the cellular level, recent in vitro studies have shown that purified *C. lanceolata*-derived compounds, including polyacetylenes, exhibit minimal toxicity toward normal human epithelial cells while demonstrating selective activity against malignant cell lines [98]. In preclinical studies, Lee et al. (2015) evaluated the acute and subchronic oral toxicity of *C. lanceolata* extract in male and female SD rats and reported no treatment-related adverse effects at doses up to 5000 mg/kg body weight, with the median lethal dose (LD_50_) estimated to exceed 5000 mg/kg [99]. These findings are further supported by broader comparative phytomedicinal assessments that classify the genus as a low-risk herbal candidate based on favorable safety records and clinical history [100].

Clinical observations have also suggested a generally acceptable safety profile for non-allergic populations. A randomized, placebo-controlled trial investigating fermented *C. lanceolata* in individuals with mild memory impairment reported no serious adverse events (ClinicalTrials.gov ID: NCT03439098). Similarly, a double-blind, randomized controlled study evaluating the effects of *C. lanceolata* extract on systolic blood pressure in prehypertensive adults identified no major safety concerns during the intervention period [66]. However, hypersensitivity reactions can occur in susceptible individuals. Hur et al. documented severe food allergies, including anaphylaxis, associated with *C. lanceolata* consumption, which were linked to increased histamine release in patients with allergic rhinitis [101]. Taken together, the available clinical evidence, along with its long history of dietary and medicinal use, suggests that *C. lanceolata* is generally well-tolerated and has not been associated with major safety concerns in the studies reported to date.

Despite the generally favorable safety profile, several important limitations remain. Considerable variability exists among studies regarding extraction procedures, processing methods, phytochemical characterization, and experimental design, making a direct comparison of findings difficult. Although numerous bioactive compounds have been identified, the specific constituents responsible for many of the reported pharmacological effects remain incompletely characterized, and the potential synergistic interactions among phytochemicals are poorly understood. Furthermore, pharmacokinetics, bioavailability, herb-drug interactions, and long-term safety data remain limited, restricting translational development. Therefore, future studies should prioritize standardized extract development, systematic structure-activity relationship investigations, comprehensive toxicity assessments, pharmacokinetic evaluations, mechanistic validation of key bioactive compounds, and large-scale clinical trials to support the safe and effective development of *C. lanceolata*-based nutraceuticals and therapeutics.

## 7. Conclusions

*Codonopsis lanceolata* continues to emerge as a versatile medicinal plant with a broad pharmacological potential across a spectrum of metabolic, inflammatory, cardiovascular, neurodegenerative, respiratory, hepatic, immune-related, and microbial disorders. Evidence accumulated from preclinical studies suggests that it has diverse bioactivities, including anti-diabetic, anti-obesity, anti-cancer, cardioprotective, hepatoprotective, neuroprotective, immunomodulatory, antimicrobial, and pulmonary-protective effects, as well as the ability to mitigate skeletal muscle atrophy and improve lactation-related dysfunction. Available evidence suggests that these therapeutic effects may involve multiple interconnected mechanisms, including the attenuation of OS and inflammation, regulation of metabolic homeostasis, modulation of immune responses, activation of neurotrophic signaling pathways, and protection against apoptosis and tissue injury. Bioactive constituents, such as triterpenoid saponins (particularly lancemasides), polysaccharides, phenolic compounds, flavonoids, and related metabolites, appear to contribute significantly to these biological activities.

A comparative evaluation of available studies indicated that the pharmacological activity of *C. lanceolata* is influenced by the extraction method, processing conditions, and phytochemical composition. Water extracts and polysaccharide-rich fractions have been predominantly associated with improvements in glucose metabolism, insulin sensitivity, and antioxidant defense, whereas ethanol, methanol, and n-butanol extracts have more frequently demonstrated anti-inflammatory, antioxidant, and anti-cancer activities. Fermentation and high-pressure processing generally enhance the phenolic content, antioxidant capacity, antimicrobial activity, and neuroprotective effects compared to non-fermented preparations. Furthermore, studies utilizing isolated compounds such as lancemaside A, echinocystic acid, and tangshenoside I have provided more direct mechanistic evidence by identifying specific molecular targets and signaling pathways. However, direct comparisons among studies remain challenging because of substantial differences in extraction procedures, phytochemical compositions, disease models, dosing regimens, and outcome measures. Therefore, although general pharmacological trends can be identified, definitive conclusions regarding the relative efficacy of different preparations require further standardized comparative investigations.

Despite these promising findings, most of the currently available evidence is derived from in vitro and animal studies, and clinical validation remains limited. Additional well-designed clinical trials are required to establish its efficacy, optimal dosage, safety profile, pharmacokinetic characteristics, and long-term therapeutic potential in humans. Future studies should prioritize phytochemical standardization, identification of active constituents, clarification of molecular targets, and the development of quality control strategies to improve reproducibility and facilitate clinical translation.

Overall, accumulating evidence suggests that *C. lanceolata* has considerable potential as a source of functional foods, nutraceuticals, and novel therapeutic agents. Continued multidisciplinary investigations integrating phytochemistry, pharmacology, toxicology, and clinical research are essential to fully elucidate the therapeutic value and support its future applications in disease prevention and treatment.

## Data Availability

No new data were created or analyzed in this study.
